# The Genetic Makeup and Expression of the Glycolytic and Fermentative Pathways Are Highly Conserved Within the *Saccharomyces* Genus

**DOI:** 10.3389/fgene.2018.00504

**Published:** 2018-11-16

**Authors:** Francine J. Boonekamp, Sofia Dashko, Marcel van den Broek, Thies Gehrmann, Jean-Marc Daran, Pascale Daran-Lapujade

**Affiliations:** ^1^Department of Biotechnology, Delft University of Technology, Delft, Netherlands; ^2^Westerdijk Institute, Utrecht, Netherlands

**Keywords:** glycolysis, promoter characterization, *Saccharomyces cerevisiae*, *Saccharomyces kudriavzevii*, Saccharomyces eubayanus, transcription factor binding sites

## Abstract

The ability of the yeast *Saccharomyces cerevisiae* to convert glucose, even in the presence of oxygen, via glycolysis and the fermentative pathway to ethanol has played an important role in its domestication. Despite the extensive knowledge on these pathways in *S. cerevisiae*, relatively little is known about their genetic makeup in other industrially relevant *Saccharomyces* yeast species. In this study we explore the diversity of the glycolytic and fermentative pathways within the *Saccharomyces* genus using *S. cerevisiae*, *Saccharomyces kudriavzevii*, and *Saccharomyces eubayanus* as paradigms. Sequencing data revealed a highly conserved genetic makeup of the glycolytic and fermentative pathways in the three species in terms of number of paralogous genes. Although promoter regions were less conserved between the three species as compared to coding sequences, binding sites for Rap1, Gcr1 and Abf1, main transcriptional regulators of glycolytic and fermentative genes, were highly conserved. Transcriptome profiling of these three strains grown in aerobic batch cultivation in chemically defined medium with glucose as carbon source, revealed a remarkably similar expression of the glycolytic and fermentative genes across species, and the conserved classification of genes into major and minor paralogs. Furthermore, transplantation of the promoters of major paralogs of *S. kudriavzevii* and *S. eubayanus* into *S. cerevisiae* demonstrated not only the transferability of these promoters, but also the similarity of their strength and response to various environmental stimuli. The relatively low homology of *S. kudriavzevii* and *S. eubayanus* promoters to their *S. cerevisiae* relatives makes them very attractive alternatives for strain construction in *S. cerevisiae*, thereby expanding the *S. cerevisiae* molecular toolbox.

## Introduction

The yeast *Saccharomyces cerevisiae* is known for its fast fermentative metabolism, which has played an important role in its domestication (Sicard and Legras, 2011). *S. cerevisiae* converts glucose to ethanol via the Embden-Meyerhof-Parnas pathway of glycolysis and the fermentative pathway, encompassing a total of 12 enzymatic steps (Barnett, 2003; Barnett and Entian, 2005). While *S. cerevisiae* can respire glucose, leading to an ATP yield of 16 moles of ATP per mole of glucose, it favors alcoholic fermentation. Indeed, even in the presence of oxygen, glucose excess triggers ethanol formation in *S. cerevisiae* and its relatives from the *Saccharomyces* genus, a phenomenon known as the Crabtree effect (De Deken, 1966; Merico et al., 2007). To sustain the energy demand for growth and maintenance despite the low ATP yield of alcoholic fermentation (2 moles of ATP per glucose molecule), the glycolytic flux in *S. cerevisiae* can easily reach fluxes of 20–25 mmoles ethanol per gram dry weight per hour (Solis-Escalante et al., 2015). This high activity of the glycolytic pathway is reflected in the remarkably high concentration of glycolytic enzymes in the cell, which can represent up to 30% of the total amount of soluble protein (Fraenkel, 2003; Carroll et al., 2011).

The genome of *S. cerevisiae* is characterized by a high genetic redundancy which can largely be attributed to a whole genome duplication event (Ohno, 1970; Wolfe and Shields, 1997). This redundancy is even more prominent among ‘metabolic’ genes and is remarkably elevated in the glycolytic and fermentative pathways of *S. cerevisiae* (Kellis et al., 2004; Kuepfer et al., 2005; Conant and Wolfe, 2007). These two pathways have been thoroughly investigated (and even established) in *S. cerevisiae* (Barnett, 2003; Van Heerden et al., 2015). With the exception of three steps that are catalyzed by single enzymes, i.e., phosphoglucose isomerase (Pgi1), fructose-bisphosphate aldolase (Fba1), and triosephosphate isomerase (Tpi1), the glycolytic, and fermentative steps are catalyzed by at least two and potentially up to seven isoenzymes for alcohol dehydrogenase (Adh). However, not all isoenzymes are equally important for the glycolytic and fermentative activity. With the notable exception of Pfk1 and Pfk2, two isoenzymes forming a heterooctamer that are equally important for the functionality of phosphofructokinase (Heinisch et al., 1991; Arvanitidis and Heinisch, 1994), for each step, a single isoenzyme is responsible for the bulk of the glycolytic and fermentative flux. These so-called major isoenzymes are encoded by major paralogs, which expression is strong and constitutive (i.e., *HXK2*, *TDH3*, *GPM1*, *ENO2*, *PYK1*, *PDC1*, *ADH1*) (Solis-Escalante et al., 2015). Because of these properties, glycolytic promoters are often used to drive gene expression in engineered strains (Peng et al., 2015). Conversely the expression of minor paralogs is, in most instances, far lower than the expression of the corresponding major paralogs and is condition-dependent (Ciriacy, 1979; Boer et al., 2003; Knijnenburg et al., 2009; Solis-Escalante et al., 2015). Following duplication events, redundant genes can have different fates. If their presence brings additional benefits to the cell, either in their native form or via neo-functionalization, the gene and its duplicate will be retained in the genome, otherwise the redundant copy will be lost (Kellis et al., 2004; Conant and Wolfe, 2008). The fact that the glycolytic and fermentative pathways still contain many paralogs that do not display obvious new functions suggests that they might increase fitness under specific conditions. For example, *PDC6* encoding a pyruvate decarboxylase with low sulfur amino acid content is specifically induced in sulfur limiting conditions (Fauchon et al., 2002; Boer et al., 2003). However, challenging this theory, it was recently shown that the simultaneous removal of all minor paralogs from the glycolytic and fermentative pathways had no detectable effect on *S. cerevisiae* physiology under a wide variety of conditions (Solis-Escalante et al., 2015).

The *Saccharomyces* genus consists of at least eight naturally occurring species which all evolved toward optimal performance in their niche, leading to different physiological characteristics (Replansky et al., 2008; Hittinger, 2013; Naseeb et al., 2017). For instance, *Saccharomyces kudriavzevii, Saccharomyces uvarum* and *Saccharomyces eubayanus* are cold-tolerant, and perform better than *S. cerevisiae* at temperatures below 20°C (Arroyo-López et al., 2010; Masneuf-Pomarède et al., 2010; Salvadó et al., 2011; Hebly et al., 2015). Strains belonging to different *Saccharomyces* species can mate and form viable hybrids, some of which play an important role in the beverage industry. For instance *Saccharomyces pastorianus*, a hybrid of *S. cerevisiae* and *S. eubayanus*, is the main lager-brewing yeast (Bond, 2009) and hybrids of *S. cerevisiae* and *S. kudriavzevii* and of *S. uvarum* and *S. eubayanus* (known as *S. bayanus*) play an important role in beer and wine fermentation (González et al., 2006; González et al., 2008; Peris et al., 2012; Nguyen and Boekhout, 2017). The cold-tolerance of *S. pastorianus* and *S. eubayanus* has indubitably promoted the selection of their hybrids with *S. cerevisiae* in cold environments (Belloch et al., 2008; Arroyo-López et al., 2010; Libkind et al., 2011).

In a recent study, using a unique yeast platform enabling the swapping of entire essential pathways, it was shown that *S. kudriavzevii* glycolytic and fermentative pathways could be transplanted in *S. cerevisiae* and could efficiently complement the native pathways. Expression of the full set of *S. kudriavzevii* orthologs in *S. cerevisiae*, expressed from *S. kudriavzevii* promoters, resulted in enzyme activities and physiological responses remarkably similar to the parental strain carrying a full set of native *S. cerevisiae* genes. However, the impact of *S. kudriavzevii* promoters on transcriptional activity in *S. cerevisiae* was not explored (Kuijpers et al., 2016). Despite *S. eubayanus* and *S. kudriavzevii* industrial importance and the availability of their full genome sequence, remarkably little is known about the genetic makeup and transcriptional regulation of the glycolytic and fermentative pathways.

To address this knowledge gap, the present study explores the diversity of the glycolytic and fermentative pathways within the genus *Saccharomyces*, using the industrially relevant yeasts *S. cerevisiae*, *S. eubayanus* and *S. kudriavzevii* as paradigms. More precisely, the presence and sequence similarity between paralogs in these three yeasts were explored. Cultivation in bioreactors combined with transcriptome analysis was used to evaluate the presence of dominant paralogs in *S. eubayanus* and *S. kudriavzevii* and to compare the expression levels of glycolytic and fermentative orthologs in their native context. Finally, we explored transferability of *S. kudriavzevii* and *S. eubayanus* promoters by monitoring their expression and context-dependency upon transplantation in *S. cerevisiae*.

## Materials and Methods

### Strains and Culture Conditions

All yeast strains used in this study are derived from the CEN.PK background (Entian and Kötter, 2007) and are listed in Table 1. Yeast cultures for transformation and genomic DNA isolation were grown in 500 mL shake flasks with 100 mL of complex, non-selective medium (YPD) containing 10 g L^−1^ Bacto Yeast extract, 20 g L^−1^ Bacto Peptone and 20 g L^−1^ glucose. Promoter regions were obtained from the strains *S. cerevisiae* CEN.PK113-7D (Van Dijken et al., 2000; Entian and Kötter, 2007; Nijkamp et al., 2012), *S. kudriavzevii* CR85 a wild isolate from oak bark (supplied by Prof. Querol and dr. Barrio, Universitat de València, Spain) (Lopes et al., 2010) and *S. eubayanus* CBS12357 (Libkind et al., 2011). The same strains were used for transcriptome analysis, with the exception of *S. cerevisiae* for which the diploid strain CEN.PK122 was used instead of the haploid CEN.PK113-7D (Entian and Kötter, 2007). All *S. cerevisiae* strains were grown at 30°C and *S. kudriavzevii* and *S. eubayanus* at 20°C in shake flasks at 200 rpm, unless different conditions are mentioned.

**Table 1:** Strains table.

Strain	Genotype	Plasmid for integration	Source
***Saccharomyces cerevisiae***			
CEN.PK113-5D	*MATa ura3-52 HIS3 LEU2 TRP1 MAL2-8c SUC2*	–	Entian and Kötter, 2007
CEN.PK113-7D	*MATa URA3 HIS3 LEU2 TRP1 MAL2-8c SUC2*	–	Van Dijken et al., 2000; Entian and Kötter, 2007; Nijkamp et al., 2012; Salazar et al., 2017
CEN.PK122	*MATa/Matα*	–	Van Dijken et al., 2000; Entian and Kötter, 2007
IMX1042	*MATa HIS3 LEU2 TRP1 MAL2-8c SUC2 ura3::(pHXK2sc-mRuby2-tENO2, URA3)*	pUDI098	This study
IMX1016	*MATa HIS3 LEU2 TRP1 MAL2-8c SUC2 ura3::(pHXK2sk-mRuby2-tENO2, URA3)*	pUDI097	This study
IMX1102	*MATa HIS3 LEU2 TRP1 MAL2-8c SUC2 ura3::(pHXK2se -mRuby2-tENO2, URA3)*	pUDI108	This study
IMX1068	*MATa HIS3 LEU2 TRP1 MAL2-8c SUC2 ura3::(pPGI1sc-mRuby2-tENO2, URA3)*	pUDI101	This study
IMX1017	*MATa HIS3 LEU2 TRP1 MAL2-8c SUC2 ura3::(pPGI1sk-mRuby2-tENO2, URA3)*	pUDI095	This study
IMX1103	*MATa HIS3 LEU2 TRP1 MAL2-8c SUC2 ura3::(pPGI1se -mRuby2-tENO2, URA3)*	pUDI109	This study
IMX1171	*MATa HIS3 LEU2 TRP1 MAL2-8c SUC2 ura3::(pPFK1sc-mRuby2-tENO2, URA3)*	pUDI121	This study
IMX1249	*MATa HIS3 LEU2 TRP1 MAL2-8c SUC2 ura3::(pPFK1sk-mRuby2-tENO2, URA3)*	pUDI126	This study
IMX1174	*MATa HIS3 LEU2 TRP1 MAL2-8c SUC2 ura3::(pPFK1se-mRuby2-tENO2, URA3)*	pUDI118	This study
IMX1175	*MATa HIS3 LEU2 TRP1 MAL2-8c SUC2 ura3::(pPFK2sc-mRuby2-tENO2, URA3)*	pUDI131	This study
IMX1176	*MATa HIS3 LEU2 TRP1 MAL2-8c SUC2 ura3::(pPFK2sk-mRuby2-tENO2, URA3)*	pUDI130	This study
IMX1177	*MATa HIS3 LEU2 TRP1 MAL2-8c SUC2 ura3::(pPFK2se-mRuby2-tENO2, URA3)*	pUDI132	This study
IMX1041	*MATa HIS3 LEU2 TRP1 MAL2-8c SUC2 ura3::(pFBA1sc-mRuby2-tENO2, URA3)*	pUDI099	This study
IMX1070	*MATa HIS3 LEU2 TRP1 MAL2-8c SUC2 ura3::(pFBA1sk-mRuby2-tENO2, URA3)*	pUDI103	This study
IMX1097	*MATa HIS3 LEU2 TRP1 MAL2-8c SUC2 ura3::(pFBA1se-mRuby2-tENO2, URA3)*	pUDI186	This study
IMX1132	*MATa HIS3 LEU2 TRP1 MAL2-8c SUC2 ura3::(pTPI1sc-mRuby2-tENO2, URA3)*	pUDI114	This study
IMX1133	*MATa HIS3 LEU2 TRP1 MAL2-8c SUC2 ura3::(pTPI1sk-mRuby2-tENO2, URA3)*	pUDI115	This study
IMX1134	*MATa HIS3 LEU2 TRP1 MAL2-8c SUC2 ura3::(pTPI1se-mRuby2-tENO2, URA3)*	pUDI116	This study
IMX1018	*MATa HIS3 LEU2 TRP1 MAL2-8c SUC2 ura3::(pTDH3sc-mRuby2-tENO2, URA3)*	pUDI094	This study
IMX1128	*MATa HIS3 LEU2 TRP1 MAL2-8c SUC2 ura3::(pTDH3sk-mRuby2-tENO2, URA3)*	pUDI110	This study
IMX1130	*MATa HIS3 LEU2 TRP1 MAL2-8c SUC2 ura3::(pTDH3se-mRuby2-tENO2, URA3)*	pUDI112	This study
IMX1043	*MATa HIS3 LEU2 TRP1 MAL2-8c SUC2 ura3::(pPGK1sc-mRuby2-tENO2, URA3)*	pUDI100	This study
IMX1019	*MATa HIS3 LEU2 TRP1 MAL2-8c SUC2 ura3::(pPGK1sk-mRuby2-tENO2, URA3)*	pUDI096	This study
IMX1069	*MATa HIS3 LEU2 TRP1 MAL2-8c SUC2 ura3::(pPGK1se-mRuby2-tENO2, URA3)*	pUDI102	This study
IMX1100	*MATa HIS3 LEU2 TRP1 MAL2-8c SUC2 ura3::(pGPM1sc-mRuby2-tENO2, URA3)*	pUDI106	This study
IMX1071	*MATa HIS3 LEU2 TRP1 MAL2-8c SUC2 ura3::(pGPM1sk-mRuby2-tENO2, URA3)*	pUDI104	This study
IMX1101	*MATa HIS3 LEU2 TRP1 MAL2-8c SUC2 ura3::(pGPM1se-mRuby2-tENO2, URA3)*	pUDI107	This study
IMX1178	*MATa HIS3 LEU2 TRP1 MAL2-8c SUC2 ura3::(pENO2sc-mRuby2-tENO2, URA3)*	pUDI122	This study
IMX1299	*MATa HIS3 LEU2 TRP1 MAL2-8c SUC2 ura3::(pENO2sk-mRuby2-tENO2, URA3)*	pUDI123	This study
IMX1180	*MATa HIS3 LEU2 TRP1 MAL2-8c SUC2 ura3::(pENO2se-mRuby2-tENO2, URA3)*	pUDI119	This study
IMX1181	*MATa HIS3 LEU2 TRP1 MAL2-8c SUC2 ura3::(pPYK1sc-Ruby2-tENO2, URA3)*	pUDI128	This study
IMX1182	*MATa HIS3 LEU2 TRP1 MAL2-8c SUC2 ura3::(pPYK1sk-mRuby2-tENO2, URA3)*	pUDI127	This study
IMX1183	*MATa HIS3 LEU2 TRP1 MAL2-8c SUC2 ura3::(pPYK1se-mRuby2-tENO2, URA3)*	pUDI129	This study
IMX1242	*MATa HIS3 LEU2 TRP1 MAL2-8c SUC2 ura3::(pPDC1sc-mRuby2-tENO2, URA3)*	pUDI161	This study
IMX1243	*MATa HIS3 LEU2 TRP1 MAL2-8c SUC2 ura3::(pPDC1sk-mRuby2-tENO2, URA3)*	pUDI162	This study
IMX1244	*MATa HIS3 LEU2 TRP1 MAL2-8c SUC2 ura3::(pPDC1se-mRuby2-tENO2, URA3)*	pUDI163	This study
IMX1245	*MATa HIS3 LEU2 TRP1 MAL2-8c SUC2 ura3::(pADH1sc-mRuby2-tENO2, URA3)*	pUDI158	This study
IMX1246	*MATa HIS3 LEU2 TRP1 MAL2-8c SUC2 ura3::(pADH1sk-mRuby2-tENO2, URA3)*	pUDI159	This study
IMX1298	*MATa HIS3 LEU2 TRP1 MAL2-8c SUC2 ura3::(pADH1se-mRuby2-tENO2, URA3)*	pUDI160	This study
IMX1099	*MATa HIS3 LEU2 TRP1 MAL2-8c SUC2 ura3::(pACT1sc-mRuby2-tENO2, URA3)*	pUDI105	This study
IMX1168	*MATa HIS3 LEU2 TRP1 MAL2-8c SUC2 ura3::(pTEF1sc-mRuby2-tENO2, URA3)*	pUDI124	This study
***Other Saccharomyces species***			
*S. kudriavzevii* CR85	*MATa/Matα*	–	Lopes et al., 2010
*S. eubayanus* CBS12357	*MATa/Matα*	–	Libkind et al., 2011

All transformations were done in *S. cerevisiae* CEN.PK113-5D using the auxotrophic marker *URA3* for selection. Synthetic medium containing 3 g L^−1^ KH_2_PO_4_, 0.5 g L ^−1^ MgSO_4_⋅7H_2_O, 5 g L^−1^ (NH_4_)_2_SO_4_, 1 mL L^−1^ of a trace element solution, and 1 mL L^−1^ of a vitamin solution was used (Verduyn et al., 1992). Synthetic medium supplemented with 20 g L^−1^ glucose (SMG) or 2% (vol/vol) ethanol (SMEtOH) was used for culture propagation where specified. For solid media 20 g L^−1^ agar was added prior to heat sterilization. For storage and propagation of plasmids *Escherichia coli* XL1-Blue (Agilent Technologies, Santa Clara, CA, United States) was used, and grown in lysogeny broth (LB) supplemented with ampicillin (100 mg L^−1^) (Bertani, 1951; Bertani, 2004). For the storage of yeast and *E. coli* strains 30% or 15% (v/v) glycerol was added to exponentially growing cultures respectively, and aliquots were stored at −80°C.

### Molecular Biology Techniques

For high fidelity PCR amplification Phusion high fidelity polymerase (Thermo Scientific, Landsmeer, Netherlands) was used according to manufacturer’s instructions. To improve efficiency of the PCR reactions, primer concentrations were decreased from 500 to 200 nM and the polymerase concentration was increased from 0.02 to 0.03 μL^−1^. PCR products were treated with 1 μL DpnI FastDigest restriction enzyme (Thermo Fisher Scientific) for 1 h at 37°C to remove residual circular templates. Afterward, the mixture was purified using GenElute^TM^ PCR Clean-Up Kit (Sigma-Aldrich, St. Louis, MO) according to manufacturer’s protocol. PCR for diagnostic purposes was done using DreamTaq PCR mastermix (Thermo Fisher Scientific) according to manufacturer’s recommendations. Primers used in this study are listed in Supplementary Tables S1, S2. PCR products were resolved on 1% agarose gel with Tris-acetate-EDTA (TAE) buffer. Genomic DNA used as template for PCR amplification of the promoter regions was isolated using YeaStar genomic DNA kit (Zymo Research, Orange, CA) according to manufacturer’s protocol. Plasmids were extracted from *E. coli* using the GenElute plasmid miniprep kit (Sigma-Aldrich) according to manufacturer’s description and eluted with miliQ water. Restriction analysis of plasmids was done using FastDigest restriction enzymes with FastDigest Green Buffer (Thermo Fisher Scientific) incubating for 30 min at 37°C according to manufacturer’s recommendations.

### Promoters, Plasmids and Yeast Strain Construction

**FIGURE 1:**
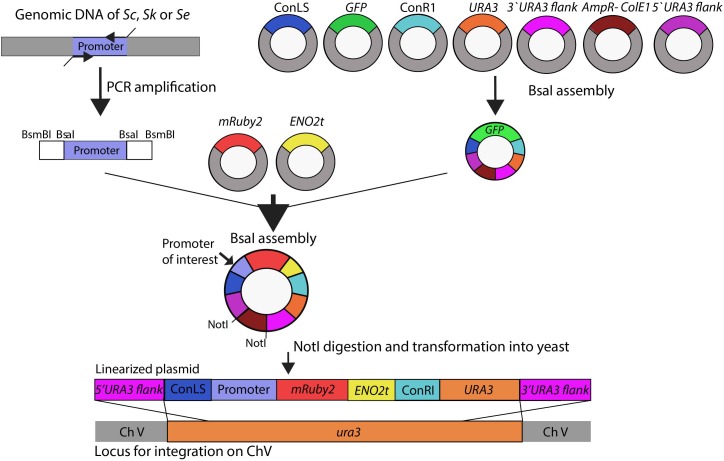
Schematic representation of the strain construction workflow. Glycolytic promoters of *Saccharomyces cerevisiae (Sc), Saccharomyces kudriavzevii (Sk)*, and *Saccharomyces eubayanus (Se)* were PCR-amplified using primers with specific BsaI flanks. First a ‘*GFP* dropout’ plasmid was assembled from the following parts containing all unique overhangs for assembly: two connectors ConLS and ConR, *URA3* marker, 5′ and 3′ *URA3* flanks and the *Amp-ColE1* containing the marker and origin of replication for *Escherichia coli*. This plasmid was used in a second round of BsaI Golden Gate assembly to replace the *GFP* fragment by the promoter of interest, *mRuby2*, and *ENO2* terminator. The resulting plasmids were linearized by NotI restriction and integrated in the *ura3* locus of *S. cerevisiae* strain IMX1076.

A schematic overview of the subsequent plasmid and strain construction steps is provided in Figure 1. Plasmids used in this study are reported in Supplementary Table S3. The *HXK2, PGI1, PFK1, PFK2, FBA1, TPI1, TDH3, PGK1, GPM1, ENO2, PYK1, PDC1* and *ADH1*, and reference *TEF1* and *ACT1* promoter regions of approximately 800 bp (see Supplementary Table S4 for exact lengths) were PCR-amplified from *S. cerevisiae* CEN.PK113-7D, *S. kudriavzevii* CR85 and *S. eubayanus* CBS 12357 genomic DNA using primers listed in Supplementary Table S1. For compatibility with Golden Gate cloning, promoter sequences were flanked with BsaI and BsmBI restriction sites introduced as primer overhangs in the PCR amplification step.

The plasmid backbone was constructed by Golden Gate assembly using the collection of part plasmids provided in the Yeast Toolkit (Lee et al., 2015). To increase the efficiency of plasmid assembly, first a *GFP* dropout plasmid pUD428 was constructed containing a *URA3* marker, AmpR selection marker, bacterial origin of replication, two connector fragments and a *GFP* gene surrounded by *URA3* upstream and downstream homology flanks (Supplementary Table S3). The correct assembly of plasmids was checked by restriction analysis. The *GFP* dropout cassette from pUD428 was subsequently replaced by the *mRuby2* gene flanked by a promoter of interest and by the *ENO2* terminator using Golden Gate cloning with BsaI. The reaction mixture was prepared with 1 μL T4 DNA ligase buffer (Thermo Fisher Scientific), 0.5 μL T7 DNA ligase (NEB New England Biolabs, Ipswich, MA), 0.5 μL FastDigest Eco31I (BsaI) (Thermo Fisher Scientific) and 10 ng of each DNA fragment. MiliQ H_2_O was added to a final volume of 10 μL. The assembly was done in a thermocycler using 25 cycles of restriction and ligation: 42°C for 2 min, 16°C for 5 min, followed by a final digestion step (60°C for 10 min) and an inactivation step (80°C for 10 min). If one of the fragments contained an internal BsaI site, the final digestion and inactivation steps were omitted. 1 μL of the assembly mix was transformed to *E. coli* (XL1-Blue) according to manufacturer’s description and plated on selective LB medium. Correct ligation of the promoter-*mRuby2*-terminator construct in this plasmid resulted in the loss of the *GFP* gene, which could be easily screened based on colony color. Additional plasmid confirmation was done by restriction analysis.

Prior to transformation into yeast, the constructed plasmids containing the promoter of interest, the *mRuby2* gene and the *ENO2* terminator were linearized by digestion with NotI (FastDigest, Thermo Fisher Scientific) according to manufacturer’s protocol for 30 min at 37°C. 400 ng of each plasmid was digested and the mixture was directly transformed to the strain CEN.PK113-5D in which the linearized plasmid was integrated in the *ura3-52* locus. Yeast transformations were done according to Gietz and Woods (Gietz and Woods, 2002). Colonies were screened by PCR (Supplementary Table S2).

### Batch Cultivation in Bioreactors

Samples for transcriptome analysis of *S. cerevisiae* (CEN.PK122), *S. kudriavzevii* (CR85) and *S. eubayanus* (CBS 12357) were obtained from aerobic batch cultures in bioreactors performed in independent duplicate. Batch cultures were performed in SMG supplemented with 0.2 g L^−1^ antifoam Emulsion C (Sigma-Aldrich). The reactors were inoculated at a starting OD_660_ of 0.3 with cells resuspended in demineralized water, which were obtained from exponentially growing shake flask cultures incubated at the same temperature and with the same medium as was used in the bioreactors (SMG). Cultures were performed in 2 L bioreactors (Applikon, Schiedam, The Netherlands) containing a 1.4 L working volume. The cultures were constantly stirred at 800 rpm, sparged with 700 mL min^−1^ dried compressed air (Linde Gas Benelux, Schiedam, The Netherlands) and maintained at 30°C for *S. cerevisiae* and 25°C for *S. kudriavzevii* and *S. eubayanus.* The culture pH was kept at 5.0 during growth on glucose by automatic addition of 2M KOH.

Extracellular metabolites were determined by high-performance liquid chromatography (HPLC) analysis using a Aminex HPX-87H ion-exchange column operated at 60°C with 5 mM H_2_SO_4_ as the mobile phase at a flow rate of 0.6 mL min^−1^ (Agilent, Santa Clara). Samples were centrifuged for 3 min at 20.000 g and the supernatant was used for analysis.

Biomass dry weight was determined in analytical duplicate by filtration of 10 mL sample on filters (pore-size 0.45 μm, Whatman/GE Healthcare Life Sciences, Little Chalfont, United Kingdom) pre-dried in a microwave oven at 360 W for 20 min, as previously described (Verduyn et al., 1992). Optical density at 660 nm (OD_660_) was determined in a Libra S11 spectrophotometer (Biocrom, Cambridge, United Kingdom). The CO_2_ and O_2_ concentration in the gas outflow was analyzed by a Rosemount NGA 2000 analyser (Baar, Switzerland), after cooling of the gas by a condenser (2°C) and drying using a PermaPure Dryer (model MD 110-8P-4; Inacom Instruments, Veenendaal, Netherlands). Sampling for transcriptome analysis was done during mid-exponential growth on glucose at a biomass concentration of approximately 1 g L^−1^. Sampling in liquid nitrogen and RNA extraction were performed as previously described (Piper et al., 2002).

### Promoter Activity Assay

Promoter activity measurement of the *mRuby2* reporter strain library was performed in 96-well plates. Precultures were grown in 12-well plates in 1.5 mL volume in a thermoshaker (Grant-bio PHMP-4, United Kingdom) with constant shaking (800 rpm) and temperature. Precultures were grown at the temperature of the subsequent plate assay (30°C or 20°C). For the first preculture YPD medium was inoculated from glycerol stocks and grown overnight till saturation. From this culture 20 μL were transferred to new 12-well plates and the strains were grown under the conditions of interest till mid-exponential phase (corresponding to OD_660_ of 3 to 5). Afterward the culture was centrifuged at 3000 g for 5 min, the supernatant was removed and cells were resuspended in fresh medium to an OD_660_ of 0.3 and transferred in volumes of 100 μL to a 96-well plate (Corning^TM^ polystyrene white/transparent bottom, Greiner Bio-One) using six replicate wells per strain. To prevent evaporation, all plates, including preculture plates, were covered with sterile polyester acrylate sealing tape (Thermo Scientific). To supply sufficient levels of oxygen throughout the cultures, small openings were created in each well with a needle. The plate assays were performed in a plate reader (TECAN infinite M200 Pro. Tecan, Männedorf, Switzerland) with constant temperature and shaking (orbital, 1 mm). Every 20 min the optical density (OD_660_) and the fluorescence using excitation and emission wavelengths 559 nm/600 nm were measured. Cultures were monitored till saturation. A non-fluorescent CEN.PK113-7D strain was taken along every run to determine the background fluorescence, as well as two reference reporter strains expressing *mRuby2* from the *TEF1* and *ACT1* promoters from *S. cerevisiae*. For every well, OD_660_ and fluorescence values from all time points during exponential growth were plotted against each other and the promoter activity was calculated as the slope of the linear regression between optical density and fluorescence.

### Flow Cytometry Analysis

mRuby2 fluorescence intensity of individual cells from cultures grown in the TECAN plate reader was determined using flow cytometry. Mid-exponential cultures from the plate reader were diluted in Isoton II (Beckman Coulter, Brea, CA) and the fluorescence intensity was determined for 10000 cells per sample on a BD FACSAriaII (Franklin Lakes, NJ) equipped with an 561 nm excitation laser and 582/15 nm emission filter. Data were analyzed using FlowJo v10.2 (FlowJo LLC). As expected from strains in which the *mRuby2* expression system is integrated in the genome the fluorescence signal was homogeneously distributed among the yeast population (Supplementary Figure S5).

### Whole Genome Sequencing

To obtain genome sequences of high quality, the strain *S. kudriavzevii* CR85 was sequenced in-house both by Illumina Miseq sequencing (Illumina, San Diego, CA) and by Oxford Nanopore Technology MinION sequencing (Oxford Nanopore Technology, Oxford, United Kingdom). Genomic DNA was isolated using the Qiagen 100/G kit (Qiagen, Hilden, Germany) and the concentration was determined using Qubit^®^ Fluorometer 2.0 (ThermoFisher Scientific). Illumina library preparation was done as described previously (Świat et al., 2017).

For Nanopore sequencing, 3 μg of genomic DNA were diluted in a total volume of 46 uL and then sheared with a g-TUBE (Covaris, Brighton, United Kingdom) to an average fragment size of 8–10 kb. The input DNA was then prepared for loading in a FLO-MIN106 flow cell with R9.4 chemistry and the 1D ligation sequencing kit (SQK-LSK108), following manufacturer’s instructions with the exception of a size selection step with 0.4x (instead of 1x) AMPure beads after the End-Repair/dA tailing module and the use of 80% (instead of 70%) ethanol for washes. Raw files generated by MinKNOW were base called using Albacore (version 1.2.5; Oxford Nanopore Technology). Reads, in fastq format, with minimum length of 1000 bp were extracted, yielding 4.15 Gigabase sequence with an average read length of 4.3 kb.

*De novo* assembly was performed using Canu (v1.4, settings: genomesize = 12m) (Koren et al., 2017) producing an 11.87 Megabase genome into 20 contigs of which 13 contigs in chromosome length plus 1 mitochondrial DNA, while 3 chromosomes consisted of 2 contigs each. The contig pairs were manually joined (with 1000 N’s between the contigs) into 3 chromosomes (chromosomes VII, XII, and XVI). Pilon (Walker et al., 2014) was then used to further correct assembly errors by aligning Illumina reads, using BWA (Li and Durbin, 2010) to the assembly using correction of only SNPs and short indels (–fix bases parameter). Gene annotations were performed using the MAKER2 annotation pipeline (version 2.31.9) (Holt and Yandell, 2011) using SNAP (version 2013–11-29) (Korf, 2004) and Augustus (version 3.2.3) (Stanke and Waack, 2003) as *ab initio* gene predictors. S288C EST and protein sequences were obtained from SGD (*Saccharomyces* Genome Database^1^) and were aligned using BLASTX (BLAST version 2.2.28+) (Camacho et al., 2009). The translated protein sequence of the final gene model was aligned using BLASTP to S288C protein Swiss-Prot database^2^. For CEN.PK113-7D and *S. eubayanus* CBS 12357 existing sequencing data was used (Baker et al., 2015; Salazar et al., 2017). The sequencing data are available at NCBI under bioproject accession number PRJNA480800.

### RNA Sequencing and Data Analysis

Library preparation and RNA sequencing were performed by Novogene Bioinformatics Technology Co., Ltd. (Yuen Long, Hong Kong). Sequencing was done with Illumina paired end 150 bp sequencing read system (PE150) using a 250∼300 bp insert strand specific library which was prepared by Novogene. For the library preparation, as described by Novogene, mRNA enrichment was done using oligo(dT) beads. After random fragmentation of the mRNA, cDNA was synthetized from the mRNA using random hexamers primers. Afterward, second strand synthesis was done by addition of a custom second strand synthesis buffer (Illumina), dNTPs, RNase H and DNA polymerase I. Finally, after terminal repair, A ligation and adaptor ligation, the double stranded cDNA library was finalized by size selection and PCR enrichment.

The sequencing data for the three strains, *S. cerevisiae* CEN.PK122, *S. kudriavzevii* CR85 and *S. eubayanus* CBS 12357 obtained by Novogene had an average read depth of 21, 24, and 24 million reads per sample, respectively. For each sample, reads were aligned to the relevant reference genome using a two-pass STAR procedure (Dobin et al., 2013). In the first pass, we assembled a splice junction database which was used to inform the second round of alignments. As paralogs in the glycolytic pathways were highly similar, we used stricter criteria for aligning and counting reads to facilitate delineation of paralogs. Introns were allowed to be between 15 and 4000 bp, and soft clipping was disabled to prevent low quality reads from being spuriously aligned. Ambiguously mapped reads were removed. Expression was quantified per transcript using htseq-count in strict intersection mode (Anders et al., 2015). As we wished to compare gene expression across genomes, where orthologs may have different gene lengths, data were normalized for gene length. Therefore the average FPKM expression counts for each gene in each species were calculated (Trapnell et al., 2010). The genomes from *S. cerevisiae* CEN.PK113-7D, *S. kudriavzevii* CR85 and *S. eubayanus* CBS 12357 were used as reference NCBI BioProject accession numbers PRJNA52955, PRJNA480800, and PRJNA264003 respectively^3^. Data are available at Gene Expression Omnibus with accession number GSE117404. CEN.PK113-7D transcriptome data is available on Gene Expression Omnibus database under accession number GSE63884.

### Comparison of DNA Sequences

Sequences from annotated glycolytic ORF and promoters of *S. cerevisiae* CEN.PK113-7D, *S. kudriavzevii* CR85 and *S. eubayanus* CBS 12357 were used for alignments with Clone Manager 9 Professional Edition, NCBI BioProject accession numbers PRJNA52955, PRJNA480800, and PRJNA264003 respectively. For the *TPI1* sequence alignment the sequences with the following accession numbers were used: CU928179 (*Z. rouxii*), HE605205 (*C. parapsilosis*), CP028453 (*Y. lipolytica*), AJ390491 (*C. albicans*), XM_002551264 (*C. tropicalis*), AJ012317 (*K. lactis*), FR839630 (*P. pastoris*) AWRI1499 (*D. bruxellensis*), XM_018355487 (*O. parapolymorpha*), CR380954 (*C. glabrata*), CP002711 (*A. gossypii*), AP014602 (*K. marxianus*), XM_001642913 (*K. polysporus*), CP000501 (*S. stipitis*), XM_002616396 (*C. lusitaniae*), and CP028714 (*E. coli*).

Alignment of these sequences was performed using multiple sequence alignment in Clustal Omega (Goujon et al., 2010; Sievers et al., 2011) and the phylogenetic trees were obtained with JalView (version 2.10.4b1) using average distance and percentage identity (Waterhouse et al., 2009).

### Statistics

Statistical analysis was performed using the software IBM SPSS statistics 23 (SPSS inc. Chicago). For transcriptome data, fluorescence data and batch culture data analysis of variance (ANOVA) with Dunnett post-hoc test was performed to test if the results for *S. kudriavzevii* and *S. eubayanus* were statistically different from *S. cerevisiae.*

## Results

### Genetic Makeup of the Glycolytic and Fermentative Pathways in *S. cerevisiae* and Its Close Relatives *S. kudriavzevii* and *S. eubayanus*

**FIGURE 2:**
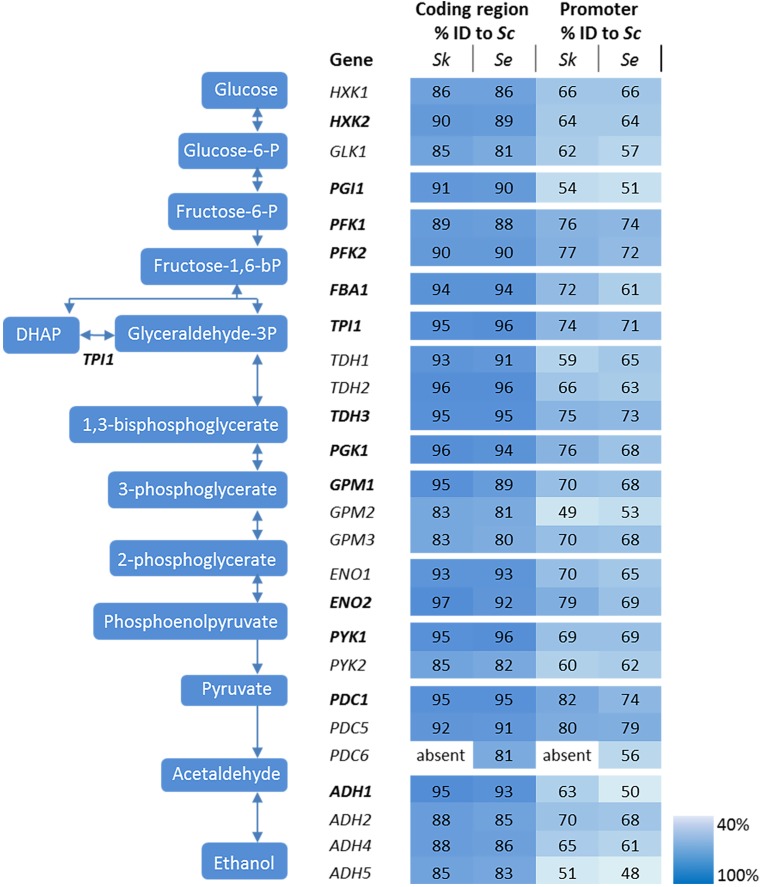
Genes and reactions involved in glycolysis and alcoholic fermentation in *S. cerevisiae* and sequence comparison between the promoters and coding regions of *S. cerevisiae* (*Sc*), *S. kudriavzevii* (*Sk*), and *S. eubayanus* (*Se*). The major paralogs in *S. cerevisiae* are represented in bold. The coding regions and promoter regions (800 bp) of *S. kudriavzevii* and *S. eubayanus* were aligned to the corresponding *S. cerevisiae* sequences and the percentage identity is indicated. *PDC6* was absent in *S. kudriavzevii.* The color scale indicates the degree of sequence identity between *S. cerevisiae* and its relatives.

The genetic makeup of pathways involved in central carbon metabolism in *S. cerevisiae* has already been well characterized, and more particularly for glycolysis and alcoholic fermentation. The ten reactions of the glycolytic pathway and the two reactions of ethanolic fermentation in *S. cerevisiae* are catalyzed by a set of 26 enzymes encoded by 26 genes (Figure 2). High quality sequences are already available for *S. cerevisiae* and *S. eubayanus* (Baker et al., 2015; Salazar et al., 2017). To explore these pathways in *S. kudriavzevii* the strain *S. kudriavzevii* CR85 was sequenced using both Illumina and Oxford Nanopore technologies (see Materials and Methods section and Supplementary Table S5). *S. cerevisiae*’s high genetic redundancy and the locations of the genes were fully mirrored in *S. kudriavzevii* and *S. eubayanus* genomes (Figure 1). The only exception was the absence of *PDC6* in *S. kudriavzevii.* While a *ScPDC6* ortholog with 81% identity was identified in *S. eubayanus*, no ortholog could be found in *S. kudriavzevii*. For all other glycolytic genes from *S. cerevisiae*, genes with 80–97% homology of the coding regions were found in *S. kudriavzevii* and *S. eubayanus* (Figure 2). Overall, genes from *S. eubayanus* were slightly more distant from their *S. cerevisiae* orthologs than genes from *S. kudriavzevii*, which is in line with earlier reports (Dujon, 2010; Shen et al., 2016; Figure 2).

In addition to the coding regions, the promoter regions were compared. Since the exact length of most promoter regions is not clearly defined, the 800 bp upstream of the coding regions were considered as promoters. Promoter sequences were substantially less conserved than the coding sequences, ranging from 43 to 78% identity when comparing *S. kudriavzevii* and *S. eubayanus* to *S. cerevisiae* promoters (Figure 2). Remarkably, some regions covering up to 45 bp were strictly conserved among the three species, whereas other parts of the promoter sequences hardly shared homology (see example of *PGK1p* on Supplementary Figure S1). As promoter regions are poorly defined, promoters shorter than 800 bp might be fully functional. Alignment with shorter regions might therefore increase the degree of homology between promoters. Alignments using 500 bp upstream the coding region only slightly increased the alignment percentages (up to 7%), mostly as a consequence of the enrichment for conserved transcription factor binding sites located between 100 and 500 bp upstream of the ORF (Harbison et al., 2004). Notably, orthologs with a relatively high or low degree of conservation between *S. cerevisiae* and *S. kudriavzevii* also displayed a similar pattern when comparing *S. eubayanus* to *S. cerevisiae.* For example, the *SkGPM2* and *SeGPM2* promoters both have a relatively low homology (49 and 53%) to the *ScGPM2* promoter, whereas the *SkPFK1* and *SePFK1* promoter have both a high degree (76 and 74%) of similarity to *ScPFK1.* Interestingly, the genes and promoters displaying a relatively low degree of homology between *S. cerevisiae* and its relatives, are homologs considered as minor in *S. cerevisiae* (for example *GPM2* and *PYK2*) (Figure 2). Blast searches did not identify additional glycolytic orthologs present in *S. eubayanus* or *S. kudriavzevii* but absent in *S. cerevisiae.*

**FIGURE 3:**
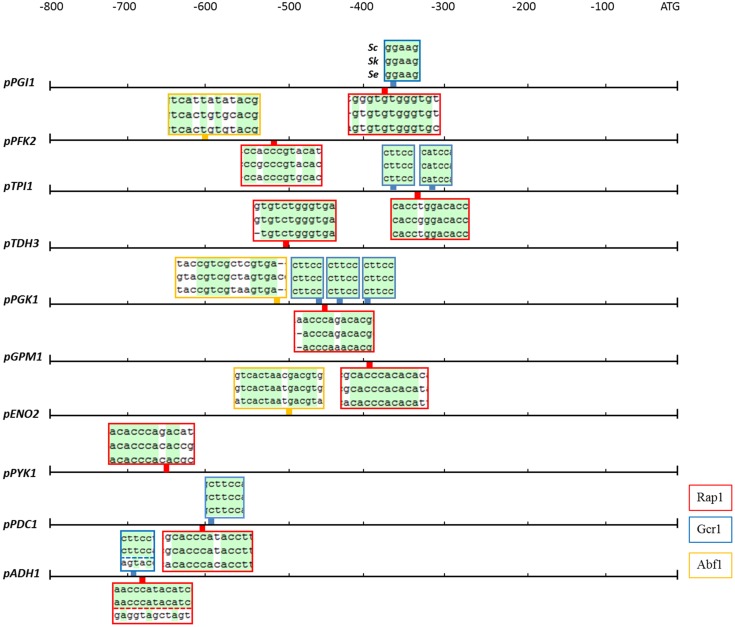
Rap1, Gcr1, and Abf1 transcription factor binding sites which are conserved in the glycolytic and fermentation promoters of *S. cerevisiae, S. kudriavzevii*, and *S. eubayanus.* The boxes indicate the location in the promoter of the binding sites for the Rap1 (red), Gcr1 (blue), and Abf1 (yellow) transcription factors which are experimentally shown to be functional in *S. cerevisiae.* The boxes contain the alignments of the three promoters at the transcription factor binding sites, conserved nucleotides are indicated in green. The Gcr1 and Rap1 sites in the *ADH1* promoter were not identified in *SeADH1p.*

The activity of a promoter strongly depends on the presence of regulatory sequences as the TATA box and other specific transcription factor binding sites. In *S. cerevisiae*, the most important glycolytic transcription factor is Gcr1, which has been experimentally shown to bind to most glycolytic promoters and to activate the expression of the corresponding genes as summarized before (Chambers et al., 1995). Gcr1 binding sites are only active when located next to DNA consensus sequences bound by Rap1 (Drazinic et al., 1996), a more pleiotropic transcription factor involved in the transcriptional regulation of a wide variety of genes including many glycolytic genes (Chambers et al., 1995). Another multifunctional transcription factor is Abf1 which binds to several glycolytic promoters (Chambers et al., 1995). With a single exception, all binding sites for Rap1, Gcr1, and Abf1 which were experimentally proven to be active in *S. cerevisiae*, were conserved in *S. kudriavzevii* and *S. eubayanus* promoter regions (Figure 3). The exception was the *SeADH1* promoter in which the Rap1 and Gcr1 site which are conserved between *S. cerevisiae* and *S. kudriavzevii* could not be identified. Together with the presence and high protein similarity of the *Se*Rap1 (82%)*, Sk*Rap1 (86%)*, Se*Gcr1 (85%), and *Sk*Gcr1 (85%), proteins with *S. cerevisiae*, these results suggested that the regulation of the glycolytic genes might be similar in the three species.

### Expression of the Glycolytic Genes During Aerobic Batch Cultivation

**FIGURE 4:**
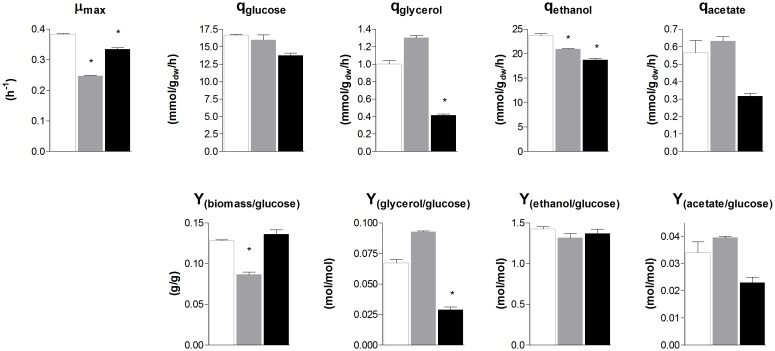
Biomass specific rates and yields of *S. cerevisiae, S. kudriavzevii*, and *S. eubayanus* batch cultivations in bioreactor. The strains were grown aerobically in synthetic medium supplemented with 20 g L^−1^ glucose. *S. cerevisiae* CEN.PK122 (white) was grown at 30°C, and *S. kudriavzevii* CR85 (gray) and *S. eubayanus* CBS 12357 (black) at 25°C. Asterisks indicate significant difference from *S. cerevisiae* (One-Way ANOVA, Dunnett *post hoc* test, *P* < 0.01).

To evaluate the similarity in glycolytic and fermentative gene expression, the transcriptome of *S. cerevisiae*, *S. kudriavzevii*, and *S. eubayanus* was compared. *S. kudriavzevii* and *S. eubayanus* are both wild isolates and both diploid (Lopes et al., 2010; Libkind et al., 2011). While many studies report the transcriptome of haploid *S. cerevisiae* strains, transcriptome data for diploid *S. cerevisiae* are scarce (Galitski et al., 1999; Li et al., 2010). To obtain comparable transcriptome datasets for the three species, the diploid CEN.PK122 strain was used. The three diploid strains were grown in aerobic batch cultures in bioreactor using minimal chemically defined medium with glucose as sole carbon source. To ensure optimal growth conditions *S. cerevisiae* was cultivated at 30°C, while its cold-tolerant relatives that have lower temperature optima were cultivated at 25°C (Arroyo-López et al., 2009; Hebly et al., 2015). Under these conditions the maximum specific growth rate of *S. cerevisiae, S. kudriavzevii*, and *S. eubayanus* was 0.38 h^−1^, 0.25 h^−1^, and 0.33 h^−1^ respectively (Figure 4). Ethanol yields were similar for the three strains, but the biomass yield of *S. kudriavzevii* was significantly lower than that of its two relatives (Figure 4), which might reflect the higher relative cost of maintenance requirements at slow growth rates (Pirt, 1982). For *S. eubayanus* we observed a lower glycerol yield as compared to its relatives, which was previously not observed under anaerobic conditions (Hebly et al., 2015).

**FIGURE 5:**
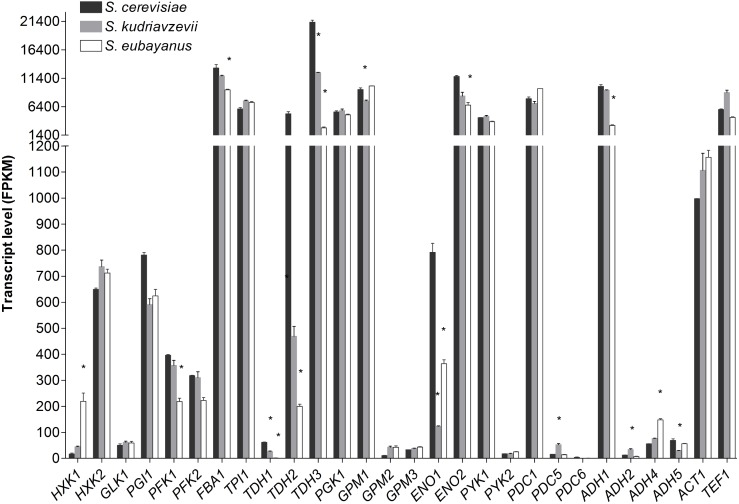
Transcript levels of the diploid strains *S. cerevisiae* (black), *S. kudriavzevii* (gray), and *S. eubayanus* (white) from two biological replicates during mid-exponential growth in aerobic batch fermentations on glucose. Asterisks indicate significant difference from *S. cerevisiae* per gene (One-Way ANOVA, Dunnett *post hoc* test, *P* < 0.01).

Transcriptome analysis of *S. cerevisiae*, *S. kudriavzevii*, and *S. eubayanus* during mid-exponential growth phase revealed a remarkable similarity between the three species (Figure 5), despite differences in culture temperature. Furthermore, the major or minor classification of paralogous genes was fully conserved between the three species (Figure 5). From the genes considered as major paralogs the *SePFK1, SeFBA1, SkTDH3, SeTDH3, SkGPM1, SeENO2*, and *SeADH1* genes displayed significantly lower expression levels as compared to *S. cerevisiae*, although only for *SeTDH3* and *SeADH1* the difference with *S. cerevisiae* was larger than 2-fold (8 and 3-fold, respectively). For the minor paralogs slightly more variability was observed. Interestingly *SeHXK1* expression was 13-fold higher than its *S. cerevisiae* ortholog. All three *TDH* genes displayed a significantly lower expression in *S. kudriavzevii* and *S. eubayanus* as compared to *S. cerevisiae.* Likewise, for *ENO1* a lower expression was observed for *SeENO1* and even lower for *SkENO1* as compared to *ScENO1.* Finally, compared to *S. cerevisiae* a ca. 3-fold higher expression was observed for *SkPDC5* and *SeADH4.*

### Optimization of Microtiter Plate Assays to Monitor Promoter Strength via Fluorescent Reporters

To explore the transferability of promoters within the *Saccharomyces* genus, the promoters of the major glycolytic and fermentative genes (indicated in bold in Figure 2) of *S. kudriavzevii* and *S. eubayanus* were functionally characterized in *S. cerevisiae*. A library of fluorescent reporter strains in which *mRuby2* expression was driven by heterologous promoters and, for comparison, by *S. cerevisiae* promoters, was constructed. To avoid bias due to gene copy number, the constructs were integrated in *S. cerevisiae* genome, at the *URA3* locus. The strains were cultured in 96-well plates, sealed with a transparent foil to prevent evaporation. Simultaneous monitoring of optical density and fluorescence revealed a premature saturation of the fluorescence signal as compared to biomass formation (Supplementary Figures S2A,B). Fluorescent proteins have a strict requirement for molecular oxygen for the synthesis of their chromophores (Tsien, 1998). The poor oxygenation of the cultures in sealed plates combined with the competition for oxygen between cellular respiration, anabolic reactions and mRuby2 maturation could explain the early saturation of the fluorescence signal. Unfortunately, this effect is rarely reported in literature and could be easily overlooked when fluorescence is measured at only one or few time points. Plate readers are widely used as method to characterize promoters with fluorescence reporters (Davis et al., 2010; Zeevi et al., 2011; Keren et al., 2013; Lee et al., 2015) however, information provided in Materials and Methods Sections are often scarce or incomplete, which makes reproduction of data by other groups difficult. To increase oxygen transfer while preventing evaporation, a small aperture was created in each well by puncturing the seal with a needle. The presence of an aperture had a strong impact on the fluorescence intensity of the cultures, enabling to monitor the cultures for a prolonged period of time (Supplementary Figure S2B). Also during growth with ethanol as sole carbon source, for which oxygen requirement is substantially increased, no premature saturation of fluorescence was observed (Supplementary Figures S2C,D). The location of the aperture in the well did not affect the fluorescence intensity (data not shown). To further evaluate the reliability of the fluorescence signal measured by the plate reader as well as the cell-to-cell heterogeneity of the fluorescence signal, measurements were also performed by flow cytometry. Comparing these data with the plate reader data revealed a very strong correlation of the fluorescence measured with these two techniques (*R*^2^ = 0,96, Supplementary Figure S3).

### Transferability and Context-Dependency of Glycolytic and Fermentative Promoters Within the *Saccharomyces* Genus

**FIGURE 6:**
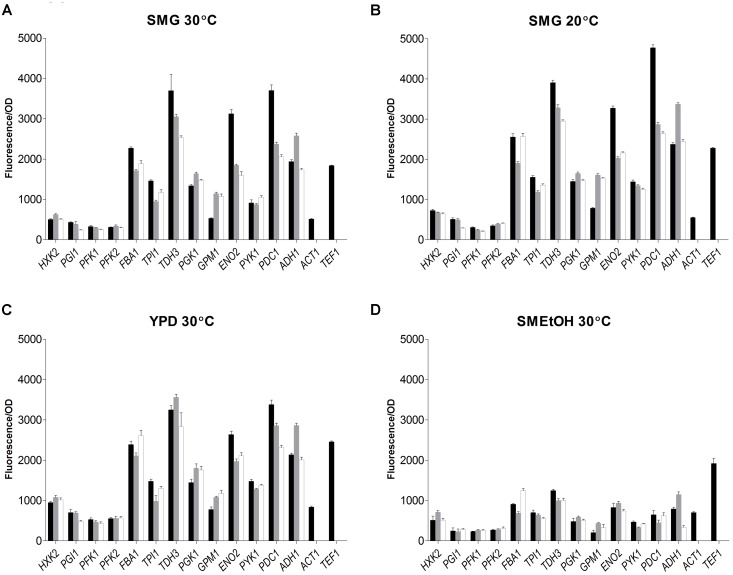
Promoter activity of the major glycolytic promoters from *S. cerevisiae* (black)*, S. kudriavzevii* (gray), and *S. eubayanus* (white) expressing *mRuby2* in *S. cerevisiae.* During exponential growth in SMG **(A)**, SMG 20°C **(B)**, YPD **(C)**, and SMEtOH **(D)** fluorescence and optical density were measured every 20 min and promoter activity was calculated as the slope of the linear regression between optical density and fluorescence. Two reference strains expressing *mRuby2* from the *ScTEF1* and *ScACT1* promoters were taken along in every plate. Error bars represent the standard deviation of the mean of six biological replicates.

The strain library grown in SMG at 30°C not only revealed that the *S. kudriavzevii* and *S. eubayanus* promoters could drive gene expression in *S. cerevisiae*, but also that their strength was remarkably similar to the strength of their *S. cerevisiae* orthologs (Figure 6). Additionally, two reporter strains expressing *mRuby2* from the constitutive *S. cerevisiae TEF1* and *ACT1* promoters were constructed and cultivated on all plates experiments. The activity of these two promoters was remarkably reproducible between independent culture replicates (Supplementary Figure S4).

While, due to high data reproducibility, expression driven by *S. kudriavzevii* or *S. eubayanus* promoters was in most cases considered statistically different from the expression led by their *S. cerevisiae* orthologs (student *t*-test, *P* < 0.01), differences in expression larger than 1.5-fold were rarely observed. Expression of *ENO2p* and *PDC1p* of *S. kudriavzevii* and *S. eubayanus* was lower than for their *S. cerevisiae* counterparts, while *SkGPM1p*, *SeGPM1p*, and *SePYK1p* led to clearly higher expression levels than their *S. cerevisiae* homologs (Figure 6). These differences were not reflected in the transcript data (Figure 5). Conversely, the differential expression of *PFK1* and *TDH3* revealed by the RNAseq analysis was also found in the promoter transplantation study at SMG 30°C. Overall similarities and differences between the three species in transcript levels were mirrored by promoter activity.

To test the condition dependency of promoter activity, strains were tested under several culture conditions. YPD was used as rich medium, and ethanol was used as gluconeogenic carbon source (SMEtOH). Since *S. kudriavzevii* and *S. eubayanus* have a lower optimum growth temperature and hexokinase from *S. kudriavzevii* has been proposed to have a lower temperature optimum as compared to *S. cerevisiae* (Gonçalves et al., 2011), the strains were also grown in SMG at 20°C. When grown in YPD and SMG at 20°C, all strains showed highly similar promoter activities as compared to cultures in SMG at 30°C even though the growth rates were different (SMG 30°C: 0.34 h^−1^, SMG 20°C: 0.15 h^−1^, YPD 30°C: 0.36 h^−1^). However, during growth on ethanol (0.13 h^−1^) promoter activity of the three species dropped tremendously as compared to glucose-grown cultures, in stark contrast with the fluorescence of the reference strains (*TEF1*p and *ACT1*p) that remained remarkably constant for all cultivation conditions. Nevertheless, also on SMEtOH *S. kudriavzevii* and *S. eubayanus* promoters showed expression levels very similar to their *S. cerevisiae* orthologs.

## Discussion

In this study we showed that the genetic makeup of the glycolytic and fermentative pathways is highly conserved among *S. cerevisiae, S. kudriavzevii*, and *S. eubayanus*. For 11 out of 12 reactions, the exact same number of paralogs was found in the three species, reflecting that species divergence took place after whole genome and post-whole genome duplications. The only exception was the absence of the minor paralog *PDC6* in the *S. kudriavzevii* CR85 genome. In agreement with this observation, the presence of a pseudogene in *S. kudriavzevii* strains IFO1802 and ZP591 consisting of only about 15% of the full *PDC6* gene length has been reported (Scannell et al., 2011). At the transcript level a strong conservation was also observed, suggesting that the classification between major and minor paralogs, confirmed in *S. cerevisiae* by mutant studies, could be extended to *S. kudriavzevii* and *S. eubayanus*. The slightly lower degree of conservation of minor paralogs (e.g., *GPM2, PYK2, ENO1, TDH1, TDH2, PDC6, ADH2, ADH4, ADH5*) is in line with the previously reported accelerated evolution of the *PYK2* and *ADH5* as compared to their *PYK1* and *ADH1* paralogs (Kellis et al., 2004).

The glycolytic pathway is known to be highly conserved compared to most other pathways (Fothergill-Gilmore and Michels, 1993; Webster, 2003). Recently it was shown that glycolytic coding regions from *E. coli* could replace the corresponding yeast genes (Kachroo et al., 2017). For promoter regions the conservation is in general lower as compared to coding regions, but a stronger conservation was found for the glycolytic promoters in the *Saccharomyces* genus than for other promoter regions (Kuang et al., 2017). Combined with the remarkable conservation of binding sites for major transcriptional regulators (i.e., Rap1, Gcr1, and Abf1), these observations suggested a very similar transcriptional regulation of glycolytic and fermentative genes across the three species. Accordingly, transcriptome data showed a remarkable conservation in expression for the majority of glycolytic and fermentative genes in their native context. It is noteworthy that transcript levels of glycolytic and fermentative genes of these three diploid species were highly similar to the transcript levels of the haploid *S. cerevisiae* CEN.PK113-7D cultivated in the same condition as the *S. cerevisiae* diploid (Solis-Escalante et al., 2015). The similarity in gene expression and the conservation of the main transcription factor binding sites in the three species suggested the possibility to introduce the promoters in *S. cerevisiae*, expecting similar regulation.

Until now a limited number of examples of heterologous glycolytic promoters driving gene expression in *S. cerevisiae* is available. Recently, it was shown that *S. kudriavzevii* glycolytic and fermentative promoters could drive gene expression in *S. cerevisiae* (Kuijpers et al., 2016). More recently, it was shown that the *ADH2* promoter of several *Saccharomyces* species could drive gene expression in *S. cerevisiae* (Harvey et al., 2018). Further, the glycolytic genes *PFK1, PFK2* and *PYK1* of the more distantly related yeast *Hanseniaspora uvarum*, expressed from their native promoters were shown to complement their *S. cerevisiae* orthologs (Langenberg et al., 2017).

**FIGURE 7:**
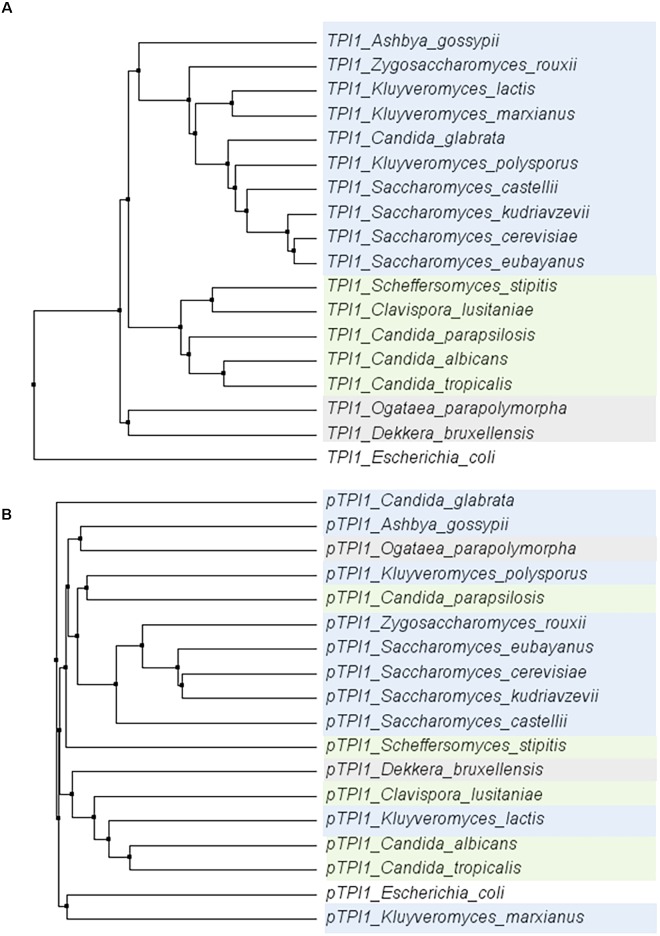
Phylogenetic trees of the alignments of the *TPI1* genes (ORF) **(A)** and promoters (800 bp) **(B)** for a set of yeast species from the *Saccharomycotina* phylum. The coding regions are strongly conserved, whereas there is hardly any conservation among promoter regions. *E. coli* was used as an outgroup. Color indicates groups as defined in Dujon (2010).

To explore the conservation of glycolytic genes in a broader context, the sequence of the *TPI1* gene was compared across a set of 18 species within the *Saccharomycotina* subphylum (Dujon, 2010). Within this subphylum, the coding region of *TPI1* was highly conserved (ranging from 64,7 to 96,3% identity to *S. cerevisiae*), while the promoters generally displayed a much weaker similarity (ranging from 28,5%–71,1% identity to *S. cerevisiae*) (Figure 7). These observations are in line with studies reporting the loss of the gene encoding the Gcr1 transcription factor and the gain of new function by Rap1 in the CTG clade yeast *Candida albicans* (Askew et al., 2009; Lavoie et al., 2010; Weirauch and Hughes, 2010). Indeed using the MEME suite motif discovery tool (Bailey et al., 2009) gave only hits for Rap1 and Gcr1 motifs in the *Saccharomyces* genus.

The present study shows the ability of all the major glycolytic promoters of *S. kudriavzevii* and *S. eubayanus* to drive gene expression in *S. cerevisiae* with similar strength and condition-dependency. Since many hybrids occur between *S. cerevisiae x S. eubayanus* and *S. cerevisiae x S. kudriavzevii*, it is not surprising that promoters are functional in *S. cerevisiae*. However, the similarity we found in promoter activities for most promoters transplanted to *S. cerevisiae* under different conditions is remarkable and indicates a strong conservation of the glycolytic regulatory mechanisms for *S. cerevisiae, S. kudriavzevii*, and *S. eubayanus* (Supplementary Figure S6). In general, these data do not correlate very well with the transcript data (Supplementary Figure S7). This can most likely be explained by the relatively low dynamic measurement range of the plate reader compared to RNAseq, differences in cultivation conditions, length of promoters, choice of site for genomic integration (Bai Flagfeldt et al., 2009) or differences in regulatory sequences in the promoters. During growth on ethanol a strong decrease in promoter activity was observed. This is in agreement with the previously reported drop in enzymatic activity of glycolysis during growth on ethanol (Peter Smits et al., 2000; Van Hoek et al., 2000).

*S. cerevisiae’s* proficiency in assembling and functionally expressing large (heterologous) pathways has propelled this yeast as preferred host for the production of complex molecules such as isoprenoids or opioids (Paddon et al., 2013; Galanie et al., 2015). However, the successful expression of these pathways depends on the availability of suitable promoters. While *S. cerevisiae* has one of the most furbished molecular toolbox, the number of constitutive and well characterized promoters remains limited. Since *S. cerevisiae’s* extremely efficient homologous recombination renders strains with repeated usage of promoter sequences genetically unstable (Manivasakam et al., 1995), this shortage of promoters presents a hurdle for extensive strain construction programs. While a lot of effort is invested in the design of synthetic promoters and transcription amplifiers (Redden and Alper, 2015; Rantasalo et al., 2016; Machens et al., 2017; Naseri et al., 2017), using slightly distant but functional orthologous promoters presents an attractive alternative (Naesby et al., 2009; Harvey et al., 2018). Usage of especially the *S. eubayanus* promoters, which are slightly more distant from *S. cerevisiae* than the *S. kudriavzevii* promoters, would reduce the length of the sequences being 100% identical to the native *S. cerevisiae* promoters. The minimum length which was found to be needed for efficient homologous recombination in *S. cerevisiae* was 30 bp with an optimal efficiency at a length of 60 bp or more (Manivasakam et al., 1995; Hua et al., 1997). In the *S. eubayanus* promoters, with one exception for the *PFK2* promoter, the longest sequence being identical to *S. cerevisiae* was found to be 34 bp. Usage of the *S. eubayanus* promoters would therefore substantially decrease the risk of instability and undesired recombination events during strain construction programs.

## Conclusion

This study brings new insight in the genetic makeup and expression of glycolytic and fermentative genes in *S. eubayanus* and *S. kudriavzevii*. It also expands the molecular toolbox for *S. cerevisiae*, but also for its two relatives, with a set of strong, constitutive promoters. Furthermore, combining Illumina and Oxford Nanopore technologies, the present study offers a high quality sequence for *S. kudriavzevii* CR85, available from NCBI (PRJNA480800). Finally, the full set of transcript levels for the three diploid strains grown in tightly controlled conditions is available via GEO (See Materials and Methods section) and can be mined to compare species-specific regulation of gene expression beyond the glycolytic and fermentative pathways.

## Author Contributions

FB, J-MD, and PD-L designed the research. FB and SD performed the experiments. MvdB and TG performed the transcriptome analysis. MvdB performed the sequence analysis. FB, SD, J-MD, and PD-L prepared the manuscript. All authors read and approved the final manuscript.

## Conflict of Interest Statement

The authors declare that the research was conducted in the absence of any commercial or financial relationships that could be construed as a potential conflict of interest.

## Footnotes

1 http://www.yeastgenome.org

2 http://www.ebi.ac.uk/swissprot/

3 https://www.ncbi.nlm.nih.gov/bioproject/

## References

[B1] Anders S. Pyl P. T. Huber W. (2015). HTSeq—a Python framework to work with high-throughput sequencing data. *Bioinformatics* 31 166–169. 10.1093/bioinformatics/btu638 25260700 PMC4287950

[B2] Arroyo-López F. N. Orlić S. Querol A. Barrio E. (2009). Effects of temperature, pH and sugar concentration on the growth parameters of *Saccharomyces cerevisiae*, *S. kudriavzevii* and their interspecific hybrid. *Int. J. Food Microbiol.* 131 120–127. 10.1016/j.ijfoodmicro.2009.01.035 19246112

[B3] Arroyo-López F. N. Pérez-Torrado R. Querol A. Barrio E. (2010). Modulation of the glycerol and ethanol syntheses in the yeast *Saccharomyces kudriavzevii* differs from that exhibited by *Saccharomyces cerevisiae* and their hybrid. *Food Microbiol.* 27 628–637. 10.1016/j.fm.2010.02.001 20510781

[B4] Arvanitidis A. Heinisch J. J. (1994). Studies on the function of yeast phosphofructokinase subunits by *in vitro* mutagenesis. *J. Biol. Chem.* 269 8911–8918. 8132627

[B5] Askew C. Sellam A. Epp E. Hogues H. Mullick A. Nantel A. (2009). Transcriptional regulation of carbohydrate metabolism in the human pathogen *Candida albicans*. *PLoS Path.* 5:e1000612. 10.1371/journal.ppat.1000612 19816560 PMC2749448

[B6] Bai Flagfeldt D. Siewers V. Huang L. Nielsen J. (2009). Characterization of chromosomal integration sites for heterologous gene expression in *Saccharomyces cerevisiae*. *Yeast* 26 545–551. 10.1002/yea.1705 19681174

[B7] Bailey T. L. Boden M. Buske F. A. Frith M. Grant C. E. Clementi L. (2009). MEME SUITE: tools for motif discovery and searching. *Nucleic Acids Res.* 37 W202–W208. 10.1093/nar/gkp335 19458158 PMC2703892

[B8] Baker E. Wang B. Bellora N. Peris D. Hulfachor A. B. Koshalek J. A. (2015). The genome sequence of *Saccharomyces eubayanus* and the domestication of lager-brewing yeasts. *Mol. Biol. Evol.* 32 2818–2831. 10.1093/molbev/msv168 26269586 PMC4651232

[B9] Barnett J. A. (2003). A history of research on yeasts 5: the fermentation pathway. *Yeast* 20 509–543. 10.1002/yea.986 12722184

[B10] Barnett J. A. Entian K. D. (2005). A history of research on yeasts 9: regulation of sugar metabolism. *Yeast* 22 835–894. 10.1002/yea.1249 16134093

[B11] Belloch C. Orlic S. Barrio E. Querol A. (2008). Fermentative stress adaptation of hybrids within the *Saccharomyces sensu* stricto complex. *Int. J. Food Microbiol.* 122 188–195. 10.1016/j.ijfoodmicro.2007.11.083 18222562

[B12] Bertani G. (1951). Studies on lysogenis I.: the mode of phage liberation by lysogenic *Escherichia coli*. *J. Bacteriol.* 62 293–300. 14888646 10.1128/jb.62.3.293-300.1951 PMC386127

[B13] Bertani G. (2004). Lysogeny at mid-twentieth century: P1. P2, and other experimental systems. *J. Bacteriol.* 186 595–600. 10.1128/JB.186.3.595-600.2004 14729683 PMC321500

[B14] Boer V. M. De Winde J. H. Pronk J. T. Piper M. D. (2003). The genome-wide transcriptional responses of *Saccharomyces cerevisiae* grown on glucose in aerobic chemostat cultures limited for carbon, nitrogen, phosphorus, or sulfur. *J. Biol. Chem.* 278 3265–3274. 10.1074/jbc.M209759200 12414795

[B15] Bond U. (2009). The genomes of lager yeasts. *Adv. Appl. Microbiol.* 69 159–182. 10.1016/S0065-2164(09)69006-7 19729094

[B16] Camacho C. Coulouris G. Avagyan V. Ma N. Papadopoulos J. Bealer K. (2009). BLAST + : architecture and applications. *BMC Bioinformatics* 10:421. 10.1186/1471-2105-10-421 20003500 PMC2803857

[B17] Carroll K. M. Simpson D. M. Eyers C. E. Knight C. G. Brownridge P. Dunn W. B. (2011). Absolute quantification of the glycolytic pathway in yeast: deployment of a complete QconCAT approach. *Mol. Cell. Proteomics* 10:M111.007633. 10.1074/mcp.M111.007633 21931151 PMC3237070

[B18] Chambers A. Packham E. A. Graham I. R. (1995). Control of glycolytic gene expression in the budding yeast (*Saccharomyces cerevisiae*). *Curr. Genet.* 29 1–9. 10.1007/BF00313187 8595651

[B19] Ciriacy M. (1979). Isolation and characterization of further cis-and trans-acting regulatory elements involved in the synthesis of glucose-repressible alcohol dehydrogenase (ADHII) in *Saccharomyces cerevisiae*. *Mol. Gen. Genet.* 176 427–431. 10.1007/BF00333107 392242

[B20] Conant G. C. Wolfe K. H. (2007). Increased glycolytic flux as an outcome of whole - genome duplication in yeast. *Mol. Syst. Biol.* 3:129. 10.1038/msb4100170 17667951 PMC1943425

[B21] Conant G. C. Wolfe K. H. (2008). Turning a hobby into a job: how duplicated genes find new functions. *Nat. Rev. Genet.* 9 938–950. 10.1038/nrg2482 19015656

[B22] Davis J. H. Rubin A. J. Sauer R. T. (2010). Design, construction and characterization of a set of insulated bacterial promoters. *Nucleic Acids Res.* 39 1131–1141. 10.1093/nar/gkq810 20843779 PMC3035448

[B23] De Deken R. (1966). The Crabtree effect: a regulatory system in yeast. *Microbiology* 44 149–156. 10.1099/00221287-44-2-149 5969497

[B24] Dobin A. Davis C. A. Schlesinger F. Drenkow J. Zaleski C. Jha S. (2013). STAR: ultrafast universal RNA-seq aligner. *Bioinformatics* 29 15–21. 10.1093/bioinformatics/bts635 23104886 PMC3530905

[B25] Drazinic C. M. Smerage J. B. López M. C. Baker H. V. (1996). Activation mechanism of the multifunctional transcription factor repressor-activator protein 1 (Rap1p). *Mol. Cell. Biol.* 16 3187–3196. 10.1128/MCB.16.6.3187 8649429 PMC231312

[B26] Dujon B. (2010). Yeast evolutionary genomics. *Nat. Rev. Genet.* 11 512–524. 10.1038/nrg2811 20559329

[B27] Entian K.-D. Kötter P. (2007). 25 Yeast genetic strain and plasmid collections. *Methods Microbiol.* 36 629–666. 10.1016/S0580-9517(06)36025-4

[B28] Fauchon M. Lagniel G. Aude J.-C. Lombardia L. Soularue P. Petat C. (2002). Sulfur sparing in the yeast proteome in response to sulfur demand. *Mol. Cell* 9 713–723. 10.1016/S1097-2765(02)00500-2 11983164

[B29] Fothergill-Gilmore L. A. Michels P. A. (1993). Evolution of glycolysis. *Prog. Biophys. Mol. Biol.* 59 105–235. 10.1016/0079-6107(93)90001-Z 8426905

[B30] Fraenkel D. G. (2003). The top genes: on the distance from transcript to function in yeast glycolysis. *Curr. Opin. Microbiol.* 6 198–201. 10.1016/S1369-5274(03)00023-7 12732312

[B31] Galanie S. Thodey K. Trenchard I. J. Interrante M. F. Smolke C. D. (2015). Complete biosynthesis of opioids in yeast. *Science* 349 1095–1100. 10.1126/science.aac9373 26272907 PMC4924617

[B32] Galitski T. Saldanha A. J. Styles C. A. Lander E. S. Fink G. R. (1999). Ploidy regulation of gene expression. *Science* 285 251–254. 10.1126/science.285.5425.251 10398601

[B33] Gietz R. D. Woods R. A. (2002). Transformation of yeast by lithium acetate/single-stranded carrier DNA/polyethylene glycol method. *Methods Enzymol.* 350 87–96. 10.1016/S0076-6879(02)50957-5 12073338

[B34] Gonçalves P. Valério E. Correia C. de Almeida J. M. Sampaio J. P. (2011). Evidence for divergent evolution of growth temperature preference in sympatric *Saccharomyces* species. *PLoS One* 6:e20739. 10.1371/journal.pone.0020739 21674061 PMC3107239

[B35] González S. S. Barrio E. Gafner J. Querol A. (2006). Natural hybrids from *Saccharomyces cerevisiae*, *Saccharomyces bayanus* and *Saccharomyces kudriavzevii* in wine fermentations. *FEMS Yeast Res.* 6 1221–1234. 10.1111/j.1567-1364.2006.00126.x 17156019

[B36] González S. S. Barrio E. Querol A. (2008). Molecular characterization of new natural hybrids of *Saccharomyces cerevisiae* and *S. kudriavzevii* in brewing. *Appl. Environ. Microbiol.* 74 2314–2320. 10.1128/AEM.01867-07 18296532 PMC2293171

[B37] Goujon M. McWilliam H. Li W. Valentin F. Squizzato S. Paern J. (2010). A new bioinformatics analysis tools framework at EMBL–EBI. *Nucleic Acids Res.* 38 W695–W699. 10.1093/nar/gkq313 20439314 PMC2896090

[B38] Harbison C. T. Gordon D. B. Lee T. I. Rinaldi N. J. Macisaac K. D. Danford T. W. (2004). Transcriptional regulatory code of a eukaryotic genome. *Nature* 431 99–104. 10.1038/nature02800 15343339 PMC3006441

[B39] Harvey C. J. Tang M. Schlecht U. Horecka J. Fischer C. R. Lin H.-C. (2018). HEx: a heterologous expression platform for the discovery of fungal natural products. *Sci. Adv.* 4:eaar5459. 10.1126/sciadv.aar5459 29651464 PMC5895447

[B40] Hebly M. Brickwedde A. Bolat I. Driessen M. R. de Hulster E. A. van den Broek M. (2015). *S. cerevisiae* × *S. eubayanus* interspecific hybrid, the best of both worlds and beyond. *FEMS Yeast Res.* 15:fov005. 10.1093/femsyr/fov005 25743788

[B41] Heinisch J. Vogelsang K. Hollenberg C. P. (1991). Transcriptional control of yeast phosphofructokinase gene expression. *FEBS Lett.* 289 77–82. 10.1016/0014-5793(91)80912-M 1832648

[B42] Hittinger C. T. (2013). *Saccharomyces* diversity and evolution: a budding model genus. *Trends Genet.* 29 309–317. 10.1016/j.tig.2013.01.002 23395329

[B43] Holt C. Yandell M. (2011). MAKER2: an annotation pipeline and genome-database management tool for second-generation genome projects. *BMC Bioinformatics* 12:491. 10.1186/1471-2105-12-491 22192575 PMC3280279

[B44] Hua S.-B. Qiu M. Chan E. Zhu L. Luo Y. (1997). Minimum length of sequence homology required for *in vivo* cloning by homologous recombination in yeast. *Plasmid* 38 91–96. 10.1006/plas.1997.1305 9339466

[B45] Kachroo A. H. Laurent J. M. Akhmetov A. Szilagyi-Jones M. McWhite C. D. Zhao A. (2017). Systematic bacterialization of yeast genes identifies a near-universally swappable pathway. *Elife.* 6:e25093. 10.7554/eLife.25093 28661399 PMC5536947

[B46] Kellis M. Birren B. W. Lander E. S. (2004). Proof and evolutionary analysis of ancient genome duplication in the yeast *Saccharomyces cerevisiae*. *Nature* 428 617–624. 10.1038/nature02424 15004568

[B47] Keren L. Zackay O. Lotan-Pompan M. Barenholz U. Dekel E. Sasson V. (2013). Promoters maintain their relative activity levels under different growth conditions. *Mol. Syst. Biol.* 9:701. 10.1038/msb.2013.59 24169404 PMC3817408

[B48] Knijnenburg T. A. Daran J.-M. G. van den Broek M. A. Daran-Lapujade P. A. de Winde J. H. Pronk J. T. (2009). Combinatorial effects of environmental parameters on transcriptional regulation in *Saccharomyces cerevisiae*: a quantitative analysis of a compendium of chemostat-based transcriptome data. *BMC Genomics* 10:53. 10.1186/1471-2164-10-53 19173729 PMC2640415

[B49] Koren S. Walenz B. P. Berlin K. Miller J. R. Bergman N. H. Phillippy A. M. (2017). Canu: scalable and accurate long-read assembly via adaptive k-mer weighting and repeat separation. *Genome Res.* 27 722–736. 10.1101/gr.215087.116 28298431 PMC5411767

[B50] Korf I. (2004). Gene finding in novel genomes. *BMC Bioinformatics* 5:59. 10.1186/1471-2105-5-59 15144565 PMC421630

[B51] Kuang Z. Pinglay S. Ji H. Boeke J. D. (2017). Msn2/4 regulate expression of glycolytic enzymes and control transition from quiescence to growth. *Elife* 6:e29938. 10.7554/eLife.29938 28949295 PMC5634782

[B52] Kuepfer L. Sauer U. Blank L. M. (2005). Metabolic functions of duplicate genes in *Saccharomyces cerevisiae*. *Genome Res.* 15 1421–1430. 10.1101/gr.3992505 16204195 PMC1240085

[B53] Kuijpers N. G. Solis-Escalante D. Luttik M. A. Bisschops M. M. Boonekamp F. J. van den Broek M. (2016). Pathway swapping: toward modular engineering of essential cellular processes. *Proc. Natl. Acad. Sci. U.S.A.* 113 15060–15065. 10.1073/pnas.1606701113 27956602 PMC5206561

[B54] Langenberg A.-K. Bink F. J. Wolff L. Walter S. von Wallbrunn C. Grossmann M. (2017). Glycolytic functions are conserved in the genome of the wine yeast *Hanseniaspora uvarum* and pyruvate kinase limits its capacity for alcoholic fermentation. *Appl. Environ. Microbiol.* 83:e01580-17. 10.1128/AEM.01580-17 28887422 PMC5666130

[B55] Lavoie H. Hogues H. Mallick J. Sellam A. Nantel A. Whiteway M. (2010). Evolutionary tinkering with conserved components of a transcriptional regulatory network. *PLoS Biol.* 8:e1000329. 10.1371/journal.pbio.1000329 20231876 PMC2834713

[B56] Lee M. E. DeLoache W. C. Cervantes B. Dueber J. E. (2015). A highly characterized yeast toolkit for modular, multipart assembly. *ACS Synth. Biol.* 4 975–986. 10.1021/sb500366v 25871405

[B57] Li B.-Z. Cheng J.-S. Ding M.-Z. Yuan Y.-J. (2010). Transcriptome analysis of differential responses of diploid and haploid yeast to ethanol stress. *J. Biotechnol.* 148 194–203. 10.1016/j.jbiotec.2010.06.013 20561546

[B58] Li H. Durbin R. (2010). Fast and accurate long-read alignment with Burrows–Wheeler transform. *Bioinformatics* 26 589–595. 10.1093/bioinformatics/btp698 20080505 PMC2828108

[B59] Libkind D. Hittinger C. T. Valério E. Gonçalves C. Dover J. Johnston M. (2011). Microbe domestication and the identification of the wild genetic stock of lager-brewing yeast. *Proc. Natl. Acad. Sci. U.S.A.* 108 14539–14544. 10.1073/pnas.1105430108 21873232 PMC3167505

[B60] Lopes C. A. Barrio E. Querol A. (2010). Natural hybrids of *S. cerevisiae x S. kudriavzevii* share alleles with European wild populations of *Saccharomyces kudriavzevii*. *FEMS Yeast Res.* 10 412–421. 10.1111/j.1567-1364.2010.00614.x 20337723

[B61] Machens F. Balazadeh S. Mueller-Roeber B. Messerschmidt K. (2017). Synthetic promoters and transcription factors for heterologous protein expression in *Saccharomyces cerevisiae*. *Front. Bioeng. Biotechnol.* 5:63. 10.3389/fbioe.2017.00063 29098147 PMC5653697

[B62] Manivasakam P. Weber S. C. McElver J. Schiestl R. H. (1995). Micro-homology mediated PCR targeting in *Saccharomyces cerevisiae*. *Nucleic Acids Res.* 23 2799–2800. 10.1093/nar/23.14.2799 7651842 PMC307107

[B63] Masneuf-Pomarède I. Bely M. Marullo P. Lonvaud-Funel A. Dubourdieu D. (2010). Reassessment of phenotypic traits for *Saccharomyces* bayanus var. uvarum wine yeast strains. *Int. J. Food Microbiol.* 139 79–86. 10.1016/j.ijfoodmicro.2010.01.038 20188428

[B64] Merico A. Sulo P. Piškur J. Compagno C. (2007). Fermentative lifestyle in yeasts belonging to the *Saccharomyces* complex. *FEBS J.* 274 976–989. 10.1111/j.1742-4658.2007.05645.x 17239085

[B65] Naesby M. Nielsen S. V. Nielsen C. A. Green T. Tange T. Ø. Simón E. (2009). Yeast artificial chromosomes employed for random assembly of biosynthetic pathways and production of diverse compounds in *Saccharomyces cerevisiae*. *Microb. Cell Fact.* 8:45. 10.1186/1475-2859-8-45 19678954 PMC2732597

[B66] Naseeb S. James S. A. Alsammar H. Michaels C. J. Gini B. Nueno-Palop C. (2017). *Saccharomyces jurei* sp. nov., Isolation and genetic identification of a novel yeast species from *Quercus robur*. *Int. J. Syst. Evol. Microbiol.* 67 2046–2052. 10.1099/ijsem.0.002013 28639933 PMC5817255

[B67] Naseri G. Balazadeh S. Machens F. Kamranfar I. Messerschmidt K. Mueller-Roeber B. (2017). Plant-derived transcription factors for orthologous regulation of gene expression in the yeast *Saccharomyces cerevisiae*. *ACS Synth. Biol.* 6 1742–1756. 10.1021/acssynbio.7b00094 28531348

[B68] Nguyen H. V. Boekhout T. (2017). Characterization of *Saccharomyces uvarum* (Beijerinck, 1898) and related hybrids: assessment of molecular markers that predict the parent and hybrid genomes and a proposal to name yeast hybrids. *FEMS Yeast Res.* 17:2. 10.1093/femsyr/fox014 28334169

[B69] Nijkamp J. F. van den Broek M. Datema E. de Kok S. Bosman L. Luttik M. A. (2012). De novo sequencing, assembly and analysis of the genome of the laboratory strain *Saccharomyces cerevisiae* CEN. PK113-7D, a model for modern industrial biotechnology. *Microb. Cell Fact.* 11:36. 10.1186/1475-2859-11-36 22448915 PMC3364882

[B70] Ohno S. (1970). *Evolution by Gene Duplication.* New York, NY: Springer 10.1007/978-3-642-86659-3

[B71] Paddon C. J. Westfall P. J. Pitera D. J. Benjamin K. Fisher K. McPhee D. (2013). High-level semi-synthetic production of the potent antimalarial artemisinin. *Nature* 496 528–532. 10.1038/nature12051 23575629

[B72] Peng B. Williams T. C. Henry M. Nielsen L. K. Vickers C. E. (2015). Controlling heterologous gene expression in yeast cell factories on different carbon substrates and across the diauxic shift: a comparison of yeast promoter activities. *Microb. Cell Fact.* 14:91. 10.1186/s12934-015-0278-5 26112740 PMC4480987

[B73] Peris D. Lopes C. A. Belloch C. Querol A. Barrio E. (2012). Comparative genomics among *Saccharomyces cerevisiae* × *Saccharomyces kudriavzevii* natural hybrid strains isolated from wine and beer reveals different origins. *BMC Genomics* 13:407. 10.1186/1471-2164-13-407 22906207 PMC3468397

[B74] Peter Smits H. Hauf J. Müller S. Hobley T. J. Zimmermann F. K. Hahn-Hägerdal B. (2000). Simultaneous overexpression of enzymes of the lower part of glycolysis can enhance the fermentative capacity of *Saccharomyces cerevisiae*. *Yeast* 16 1325–1334. 10.1002/1097-0061(200010)16:14<1325::AID-YEA627>3.0.CO;2-E 11015729

[B75] Piper M. D. Daran-Lapujade P. Bro C. Regenberg B. Knudsen S. Nielsen J. (2002). Reproducibility of oligonucleotide microarray transcriptome analyses an interlaboratory comparison using chemostat cultures of *Saccharomyces cerevisiae*. *J. Biol. Chem.* 277 37001–37008. 10.1074/jbc.M204490200 12121991

[B76] Pirt S. (1982). Maintenance energy: a general model for energy-limited and energy-sufficient growth. *Arch. Microbiol.* 133 300–302. 10.1007/BF00521294 7171288

[B77] Rantasalo A. Czeizler E. Virtanen R. Rousu J. Lähdesmäki H. Penttilä M. (2016). Synthetic transcription amplifier system for orthogonal control of gene expression in *Saccharomyces cerevisiae*. *PLoS One* 11:e0148320. 10.1371/journal.pone.0148320 26901642 PMC4762949

[B78] Redden H. Alper H. S. (2015). The development and characterization of synthetic minimal yeast promoters. *Nat. Common.* 6:7810. 10.1038/ncomms8810 26183606 PMC4518256

[B79] Replansky T. Koufopanou V. Greig D. Bell G. (2008). Saccharomyces sensu stricto as a model system for evolution and ecology. *Trends Ecol. Evol.* 23 494–501. 10.1016/j.tree.2008.05.005 18656281

[B80] Salazar A. N. Gorter de Vries A. R. van den Broek M. Wijsman M. de la Torre Cortés P. Brickwedde A. (2017). Nanopore sequencing enables near-complete de novo assembly of *Saccharomyces cerevisiae* reference strain CEN. PK113-7D. *FEMS Yeast Res.* 17:fox074. 10.1093/femsyr/fox074 28961779 PMC5812507

[B81] Salvadó Z. Arroyo-López F. Guillamón J. Salazar G. Querol A. Barrio E. (2011). Temperature adaptation markedly determines evolution within the genus *Saccharomyces*. *Appl. Environ. Microbiol.* 77 2292–2302. 10.1128/AEM.01861-10 21317255 PMC3067424

[B82] Scannell D. R. Zill O. A. Rokas A. Payen C. Dunham M. J. Eisen M. B. (2011). The awesome power of yeast evolutionary genetics: new genome sequences and strain resources for the *Saccharomyces sensu* stricto genus. *G*3 1 11–25. 10.1534/g3.111.000273 22384314 PMC3276118

[B83] Shen X.-X. Zhou X. Kominek J. Kurtzman C. P. Hittinger C. T. Rokas A. (2016). Reconstructing the backbone of the *Saccharomycotina* yeast phylogeny using genome-scale data. *G*3 6 3927–3939. 10.1534/g3.116.034744 27672114 PMC5144963

[B84] Sicard D. Legras J.-L. (2011). Bread, beer and wine: yeast domestication in the *Saccharomyces sensu* stricto complex. *C. R. Biol.* 334 229–236. 10.1016/j.crvi.2010.12.016 21377618

[B85] Sievers F. Wilm A. Dineen D. Gibson T. J. Karplus K. Li W. (2011). Fast, scalable generation of high-quality protein multiple sequence alignments using Clustal Omega. *Mol. Syst. Biol.* 7:539. 10.1038/msb.2011.75 21988835 PMC3261699

[B86] Solis-Escalante D. Kuijpers N. G. Barrajon-Simancas N. van den Broek M. Pronk J. T. Daran J. M. (2015). A minimal set of glycolytic genes reveals strong redundancies in *Saccharomyces cerevisiae* central metabolism. *Eukaryot. Cell* 14 804–816. 10.1128/EC.00064-15 26071034 PMC4519752

[B87] Stanke M. Waack S. (2003). Gene prediction with a hidden Markov model and a new intron submodel. *Bioinformatics* 19 ii215–ii225. 10.1093/bioinformatics/btg1080 14534192

[B88] Świat M. A. Dashko S. den Ridder M. Wijsman M. van der Oost J. Daran J.-M. (2017). FnCpf1: a novel and efficient genome editing tool for *Saccharomyces cerevisiae*. *Nucleic Acids Res.* 45 12585–12598. 10.1093/nar/gkx1007 29106617 PMC5716609

[B89] Trapnell C. Williams B. A. Pertea G. Mortazavi A. Kwan G. Van Baren M. J. (2010). Transcript assembly and quantification by RNA-Seq reveals unannotated transcripts and isoform switching during cell differentiation. *Nat. Biotechnol.* 28 511–515. 10.1038/nbt.1621 20436464 PMC3146043

[B90] Tsien R. Y. (1998). The green fluorescent protein. *Annu. Rev. Biochem.* 67 509–544. 10.1146/annurev.biochem.67.1.509 9759496

[B91] Van Dijken J. Bauer J. Brambilla L. Duboc P. Francois J. Gancedo C. (2000). An interlaboratory comparison of physiological and genetic properties of four *Saccharomyces cerevisiae* strains. *Enzyme Microb. Technol.* 26 706–714. 10.1016/S0141-0229(00)00162-9 10862876

[B92] Van Heerden J. H. Bruggeman F. J. Teusink B. (2015). Multi-tasking of biosynthetic and energetic functions of glycolysis explained by supply and demand logic. *Bioessays* 37 34–45. 10.1002/bies.201400108 25350875

[B93] Van Hoek P. Van Dijken J. P. Pronk J. T. (2000). Regulation of fermentative capacity and levels of glycolytic enzymes in chemostat cultures of *Saccharomyces cerevisiae*. *Enzyme Microb. Technol.* 26 724–736. 10.1016/S0141-0229(00)00164-2 10862878

[B94] Verduyn C. Postma E. Scheffers W. A. Van Dijken J. P. (1992). Effect of benzoic acid on metabolic fluxes in yeasts: a continuous-culture study on the regulation of respiration and alcoholic fermentation. *Yeast* 8 501–517. 10.1002/yea.320080703 1523884

[B95] Walker B. J. Abeel T. Shea T. Priest M. Abouelliel A. Sakthikumar S. (2014). Pilon: an integrated tool for comprehensive microbial variant detection and genome assembly improvement. *PLoS One* 9:e112963. 10.1371/journal.pone.0112963 25409509 PMC4237348

[B96] Waterhouse A. M. Procter J. B. Martin D. M. Clamp M. Barton G. J. (2009). Jalview Version 2—a multiple sequence alignment editor and analysis workbench. *Bioinformatics* 25 1189–1191. 10.1093/bioinformatics/btp033 19151095 PMC2672624

[B97] Webster K. A. (2003). Evolution of the coordinate regulation of glycolytic enzyme genes by hypoxia. *J. Exp. Biol.* 206 2911–2922. 10.1242/jeb.00516 12878660

[B98] Weirauch M. T. Hughes T. R. (2010). Conserved expression without conserved regulatory sequence: the more things change, the more they stay the same. *Trends Genet.* 26 66–74. 10.1016/j.tig.2009.12.002 20083321

[B99] Wolfe K. H. Shields D. C. (1997). Molecular evidence for an ancient duplication of the entire yeast genome. *Nature* 387 708–713. 10.1038/42711 9192896

[B100] Zeevi D. Sharon E. Lotan-Pompan M. Lubling Y. Shipony Z. Raveh-Sadka T. (2011). Compensation for differences in gene copy number among yeast ribosomal proteins is encoded within their promoters. *Genome Res.* 21 2114–2128. 10.1101/gr.119669.110 22009988 PMC3227101

