# Supplementary material for: The Genetic Makeup and Expression of the Glycolytic and Fermentative Pathways Are Highly Conserved Within the Saccharomyces Genus
Source: Front Genet. 2018 Nov 16;9:504. doi: 10.3389/fgene.2018.00504 (PMC6250768; doi:10.3389/fgene.2018.00504)
Supplement: Supplementary file 1 [file Table_1.DOCX]

Supplementary Material

**The genetic makeup and expression of the glycolytic and fermentative pathways are highly conserved within the *Saccharomyces* genus**

**Francine J. Boonekamp, Sofia Dashko, Marcel van den Broek, Thies Gehrmann, Jean-Marc Daran, Pascale Daran-Lapujade***

*** Correspondence:** [p.a.s.daran-lapujade@tudelft.nl](mailto:p.a.s.daran-lapujade@tudelft.nl)

# Table S1

Primer for the amplification of promoters with BsmBI and BsaI restriction site flanks for Golden Gate cloning.

| Promoter Primer sequence | |
| --- | --- |
| *pHXK2sc fw* | AAGCATCGTCTCATCGGTCTCAAACGCTGGTAAAGTACAGCTACATTC |
| *pHXK2sc rev* | TTATGCCGTCTCAGGTCTCACATATTTATTTAATTAGCGTACTTATTATGTGTGG |
| *pHXK2sk fw* | GCATCGTCTCATCGGTCTCAAACGACGATCACACACATGCGTTC |
| *pHXK2sk rev* | ATGCCGTCTCAGGTCTCACATATTGTTATTTATTATTGCGTTTAGTATATGTAG |
| *pHXK2se fw* | AAGCATCGTCTCATCGGTCTCAAACGCGCGCACGCATTCATCC |
| *pHXK2se rev* | TTATGCCGTCTCAGGTCTCACATATTTTTTTTATGAGTATGTGTAGTATGTGTACAAAAG |
| *pPGI1sc fw* | AAGCATCGTCTCATCGGTCTCAAACGTATTCTTAGTGGATAACATGCG |
| *pPGI1sc rev* | TTATGCCGTCTCAGGTCTCACATATTTTAGGCTGGTATCTTGATTC |
| *pPGI1sk fw* | GCATCGTCTCATCGGTCTCAAACGACCTCATTGGTTCAGTACTTTTC |
| *pPGI1sk rev* | ATGCCGTCTCAGGTCTCACATATTTTAGGTTAGTATCTTGACTTTAAATC |
| *pPGI1se fw* | AAGCATCGTCTCATCGGTCTCAAACGCAAGAACGATTGCTTTTGTTGG |
| *pPGI1se rev* | TTATGCCGTCTCAGGTCTCACATATTTTAGGCTAATACTTTGCTTTTCAATC |
| *pPFK1sc fw* | AAGCATCGTCTCATCGGTCTCAAACGCGGCTAGTAAAAAAGAAAATTAATATCTCATTAAC |
| *pPFK1sc rev* | TTATGCCGTCTCAGGTCTCACATACTTTGATATGATTTTGTTTCAGATTTTTTATATAAAAGC |
| *pPFK1sk fw* | AAGCATCGTCTCATCGGTCTCAAACGCTTTCTCTTCAGCGCTATGC |
| *pPFK1sk rev* | TTATGCCGTCTCAGGTCTCACATACTTTGATAATGGTTTGTTTTCAGATTATTTTTTATAG |
| *pPFK1se fw* | AAGCATCGTCTCATCGGTCTCAAACGTAACCGTGCTCCTGGATAGC |
| *pPFK1se rev* | TTATGCCGTCTCAGGTCTCACATAATTTGTTAGTTGTTTCGTTTTAGATTATTTTG |
| *pPFK2sc fw* | AAGCATCGTCTCATCGGTCTCAAACGCCATTCTCTGCTGCTTTGTTG |
| *pPFK2sc rev* | TTATGCCGTCTCAGGTCTCACATATGCGTATGGTTAGTTCTTGGC |
| *pPFK2sk fw* | AAGCATCGTCTCATCGGTCTCAAACGGTGTGAACGCTGCTCATAGC |
| *pPFK2sk rev* | TTATGCCGTCTCAGGTCTCACATATGCGTATGGTTATTTCTTGGCC |
| *pPFK2se fw* | AAGCATCGTCTCATCGGTCTCAAACGGTTCCCAGCCGATTTGTTGG |
| *pPFK2se rev* | TTATGCCGTCTCAGGTCTCACATATGCGTATGGTTAGTTCTTGGCC |
| *pFBA1sc fw* | AAGCATCGTCTCATCGGTCTCAAACGCAATACCAGCCTTCCAACTTC |
| *pFBA1sc rev* | TTATGCCGTCTCAGGTCTCACATATTTGAATATGTATTACTTGGTTATGG |
| *pFBA1sk fw* | AAGCATCGTCTCATCGGTCTCAAACGAACAACAATGCCAACCC |
| *pFBA1sk rev* | TTATGCCGTCTCAGGTCTCACATATTTGAATATGTGTTACTTGTGTATG |
| *pFBA1se fw* | AAGCATCGTCTCATCGGTCTCAAACGCTTTTCCCATGTTTCCAATGCC |
| *pFBA1se rev* | TTATGCCGTCTCAGGTCTCACATATTTGTATATGTTTTACTTGTGTATGG |
| *pTPI1sc fw* | AAGCATCGTCTCATCGGTCTCAAACGACCCAGAGATGTTGTTGTCC |
| *pTPI1sc rev* | TTATGCCGTCTCAGGTCTCACATATTTTAGTTTATGTATGTGTTTTTTGTAG |
| *pTPI1sk fw* | AAGCATCGTCTCATCGGTCTCAAACGGGATGTTGTTGTTCTTGTCAC |
| *pTPI1sk rev* | TTATGCCGTCTCAGGTCTCACATATTTTAATGTATGTATGTATATGAGATTTTTGTAG |
| *pTPI1se fw* | AAGCATCGTCTCATCGGTCTCAAACGGGATGTCGTTGTTCTTGTTAC |
| *pTPI1se rev* | TTATGCCGTCTCAGGTCTCACATATTTTAGTGTATGTGTATGTGTGTTTG |
| *pTDH3sk fw* | AAGCATCGTCTCATCGGTCTCAAACGGCGAATTTTTACTAACCTTGAATG |
| *pTDH3sk rev* | TTATGCCGTCTCAGGTCTCACATATGTGTTTATTTGTGTGTGTTTATTC |
| *pTDH3se fw* | AAGCATCGTCTCATCGGTCTCAAACGGTCAATAACAAGAAATTTAATGACGC |
| *pTDH3se rev* | TTATGCCGTCTCAGGTCTCACATATTTTATTGTATGTGTGTGTGTTTGAAACTA |
| *pPGK1sc fw* | AAGCATCGTCTCATCGGTCTCAAACGTATTTTAGATTCCTGACTTCAACTC |
| *pPGK1sc rev* | TTATGCCGTCTCAGGTCTCACATATGTTTTATATTTGTTGTAAAAAGTAGATAATTAC |
| *pPGK1sk fw* | GCATCGTCTCATCGGTCTCAAACGTTAGCTTCAACTCAAGATGTACAG |
| *pPGK1sk rev* | ATGCCGTCTCAGGTCTCACATATGTTTTATATTTGTTGTAAAAAGTAGATAATTAC |
| *pPGK1se fw* | AAGCATCGTCTCATCGGTCTCAAACGGCTTCAATTCAAGATACACAGATATAC |
| *pPGK1se rev* | TTATGCCGTCTCAGGTCTCACATATGTTTTATATTTGTTGCAAAAAGTAG |
| *pGPM1sc fw* | AAGCATCGTCTCATCGGTCTCAAACGGTGATACTTTGACAGGAGC |
| *pGPM1sc rev* | TTATGCCGTCTCAGGTCTCACATATATTGTAATATGTGTGTTTGTTTGG |
| *pGPM1sk fw* | AAGCATCGTCTCATCGGTCTCAAACGAAGGTAGCAACAAGAGAGTAG |
| *pGPM1sk rev* | TTATGCCGTCTCAGGTCTCACATATTTTGTAATTTACGTTTGTGTGC |
| *pGPM1se fw* | AAGCATCGTCTCATCGGTCTCAAACGTAAACCTGATCTTTCACCTCAGTAAC |
| *pGPM1se rev* | TTATGCCGTCTCAGGTCTCACATATTCTGTGATATATGTGTGTATGCTTAC |
| *pENO2sc fw* | AAGCATCGTCTCATCGGTCTCAAACGGGATGATGAAAACACTAAACGAAG |
| *pENO2sc rev* | TTATGCCGTCTCAGGTCTCACATATATTATTGTATGTTATAGTATTAGTTGCTTGG |
| *pENO2sk fw* | AAGCATCGTCTCATCGGTCTCAAACGGATGATAAAATCACTAAACGAAGAAC |
| *pENO2sk rev* | TTATGCCGTCTCAGGTCTCACATATATTATTGTATGATATAGTATTAGTTGCTTGG |
| *pENO2se fw* | AAGCATCGTCTCATCGGTCTCAAACGCCAAGAAGATGCCGGCTAC |
| *pENO2se rev* | TTATGCCGTCTCAGGTCTCACATATATTATTGTTTGATATAGTATTAGTTGCTTGGT |
| *pPYK1sc fw* | AAGCATCGTCTCATCGGTCTCAAACGCCCTGGTCAAACTTCAGAAC |
| *pPYK1sc rev* | TTATGCCGTCTCAGGTCTCACATATGTGATGATGTTTTATTTGTTTTGATTG |
| *pPYK1sk fw* | AAGCATCGTCTCATCGGTCTCAAACGAGCAGCAGTTGCAAAATTAGC |
| *pPYK1sk rev* | TTATGCCGTCTCAGGTCTCACATATGTGATGATGTTTTATTTGTTTTGATTAGTG |
| *pPYK1se fw* | AAGCATCGTCTCATCGGTCTCAAACGTGTAAATACCGGTTTTAGCC |
| *pPYK1se rev* | TTATGCCGTCTCAGGTCTCACATATGTGATGATGTTTTATTTGTTTTG |
| *pPDC1sc fw* | AAGCATCGTCTCATCGGTCTCAAACGCATGCGACTGGGTGAGCATATG |
| *pPDC1sc rev* | TTATGCCGTCTCAGGTCTCACATATTTGATTGATTTGACTGTGTTATTTTGCG |
| *pPDC1sk fw* | AAGCATCGTCTCATCGGTCTCAAACGGGGTAGCGACGCGTGGG |
| *pPDC1sk rev* | TTATGCCGTCTCAGGTCTCACATATATGATTGTTTTGACTGTGCTATTTTGTGTG |
| *pPDC1se fw* | AAGCATCGTCTCATCGGTCTCAAACGGATGAAGTGACGCGCGCCC |
| *pPDC1se rev* | TTATGCCGTCTCAGGTCTCACATATATGGTTGTTTTGACTGTGTTATTTTGTGTGAG |
| *pADH1sc fw* | AAGCATCGTCTCATCGGTCTCAAACGAAGTCCAATGCTAGTAGAGAAG |
| *pADH1sc rev* | TTATGCCGTCTCAGGTCTCACATATGTATATGAGATAGTTGATTGTATGC |
| *pADH1sk fw* | AAGCATCGTCTCATCGGTCTCAAACGCACTCCCAAATAATCAAGGGGGTGTTAC |
| *pADH1sk rev* | TTATGCCGTCTCAGGTCTCACATATGTATATGAGATAGTTGATTGTATGCTTGGTATAGC |
| *pADH1se fw* | AAGCATCGTCTCATCGGTCTCAAACGTAAATTGCCAAGCAGCGTGAC |
| *pADH1se rev* | TTATGCCGTCTCAGGTCTCACATATGTATATGAGATAGTTGATTGTATGCTTGGTAC |
| *pACT1sc fw* | AAGCATCGTCTCATCGGTCTCAAACGCCATGGCTAGACAAATCAAGG |
| *pACTsc rev* | TTATGCCGTCTCAGGTCTCACATATTAATTCAGTAAATTTTCGATCTTGGG |

# Table S2

Primers to check integration of promoter-mRuby2-tENO2 construct in *ura3* locus.

| Primer | Sequence | Source |
| --- | --- | --- |
| *URA3* 5' barcode Yeast toolkit | GTAATGTTATCCATGTGGGC |  |
| *URA3* upstream flank | ATTCCAACTAATGAGATGGAATCG | This study |
| *URA3* downstream flank | CCAGCCCATATCCAACTTCC | This study |
| *URA3* 3' barcode Yeast toolkit | AGAGCACTTGAATCCACTGC |  |

# Table S3

Plasmids used in this study.

| Plasmid | Content | Origin |
| --- | --- | --- |
| pYTK013 | *pTEF1sc* | Lee *et al.* 2015 (Lee et al., 2015) |
| pYTK009 | *pTDH3sc* | Lee *et al.* 2015 (Lee et al., 2015) |
| pYTK034 | *mRuby2* | Lee *et al.* 2015 (Lee et al., 2015) |
| pYTK055 | *tENO2sc* | Lee *et al.* 2015 (Lee et al., 2015) |
| pYTK002 | ConLS | Lee *et al.* 2015 (Lee et al., 2015) |
| pYTK047 | *GFP* dropout | Lee *et al.* 2015 (Lee et al., 2015) |
| pYTK067 | ConR1 | Lee *et al.* 2015 (Lee et al., 2015) |
| pYTK074 | *URA3* | Lee *et al.* 2015 (Lee et al., 2015) |
| pYTK086 | *URA3* 3' Homology | Lee *et al.* 2015 (Lee et al., 2015) |
| pYTK089 | *AmpR*-ColE1 | Lee *et al.* 2015 (Lee et al., 2015) |
| pYTK092 | *URA3* 5' Homology | Lee *et al.* 2015 (Lee et al., 2015) |
| pUD428 | GFP dropout plasmid assembled from  pYTK2, 47, 67, 74, 86, 89, 92 | This study, used as backbone to construct all plasmids below |
| pUDI098 | *pHXK2 sc-mRuby2-tENO2* | This study |
| pUDI097 | *pHXK2 sk-mRuby2-tENO2* | This study |
| pUDI108 | *pHXK2 se-mRuby2-tENO2* | This study |
| pUDI101 | *pPGI1 sc-mRuby2-tENO2* | This study |
| pUDI095 | *pPGI1 sk-mRuby2-tENO2* | This study |
| pUDI109 | *pPGI1 se-mRuby2-tENO2* | This study |
| pUDI121 | *pPFK1 sc-mRuby2-tENO2* | This study |
| pUDI126 | *pPFK1 sk-mRuby2-tENO2* | This study |
| pUDI118 | *pPFK1 se-mRuby2-tENO2* | This study |
| pUDI131 | *pPFK2 sc-mRuby2-tENO2* | This study |
| pUDI130 | *pPFK2 sk-mRuby2-tENO2* | This study |
| pUDI132 | *pPFK2 se-mRuby2-tENO2* | This study |
| pUDI099 | *pFBA1 sc-mRuby2-tENO2* | This study |
| pUDI103 | *pFBA1 sk-mRuby2-tENO2* | This study |
| pUDI186 | *pFBA1 se-mRuby2-tENO2* | This study |
| pUDI114 | *pTPI1 sc-mRuby2-tENO2* | This study |
| pUDI115 | *pTPI1 sk-mRuby2-tENO2* | This study |
| pUDI116 | *pTPI1 se-mRuby2-tENO2* | This study |
| pUDI094 | *pTDH3 sc-mRuby2-tENO2* | This study |
| pUDI110 | *pTDH3 sk-mRuby2-tENO2* | This study |
| pUDI112 | *pTDH3 se-mRuby2-tENO2* | This study |
| pUDI100 | *pPGK1 sc-mRuby2-tENO2* | This study |
| pUDI096 | *pPGK1 sk-mRuby2-tENO2* | This study |
| pUDI102 | *pPGK1 se-mRuby2-tENO2* | This study |
| pUDI106 | *pGPM1 sc-mRuby2-tENO2* | This study |
| pUDI104 | *pGPM1 sk-mRuby2-tENO2* | This study |
| pUDI107 | *pGPM1 se-mRuby2-tENO2* | This study |
| pUDI122 | *pENO2 sc-mRuby2-tENO2* | This study |
| pUDI123 | *pENO2 sk-mRuby2-tENO2* | This study |
| pUDI119 | *pENO2 se-mRuby2-tENO2* | This study |
| pUDI128 | *pPYK1 sc-mRuby2-tENO2* | This study |
| pUDI127 | *pPYK1 sk-mRuby2-tENO2* | This study |
| pUDI129 | *pPYK1 se-mRuby2-tENO2* | This study |
| pUDI161 | *pPDC1 sc-mRuby2-tENO2* | This study |
| pUDI162 | *pPDC1 sk-mRuby2-tENO2* | This study |
| pUDI163 | *pPDC1 se-mRuby2-tENO2* | This study |
| pUDI158 | *pADH1 sc-mRuby2-tENO2* | This study |
| pUDI159 | *pADH1 sk-mRuby2-tENO2* | This study |
| pUDI160 | *pADH1 se-mRuby2-tENO2* | This study |
| pUDI124 | *pTEF1 sc-mRuby2-tENO2* | This study |
| pUDI105 | *pACT1 sc-mRuby2-tENO2* | This study |

# Table S4

Length in base pairs of promoters used in this study.

|  | *S. cerevisiae* | *S. kudriavzevii* | *S. eubayanus* |
| --- | --- | --- | --- |
| *pHXK2* | 800 | 800 | 800 |
| *pPGI1* | 800 | 800 | 800 |
| *pPFK1* | 800 | 805 | 800 |
| *pPFK2* | 800 | 798 | 800 |
| *pFBA1* | 793 | 800 | 781 |
| *pTPI1* | 798 | 801 | 796 |
| *pTDH1* | 800 | 800 | 800 |
| *pTDH3* | 689 | 800 | 800 |
| *pPGK1* | 778 | 764 | 796 |
| *pGPM1* | 800 | 800 | 503 |
| *pENO2* | 800 | 800 | 800 |
| *pPYK1* | 811 | 800 | 807 |
| *pPDC1* | 800 | 800 | 800 |
| *pADH1* | 800 | 845 | 800 |

# Table S5: *S. kudriavzevii* sequencing

The Nanopore assembly of the S. kudriavzevii CR85 sequencing data resulted in only 20 contigs, which represents a near 73-fold reduction in the number of contigs and captured an additional 200 kb as compared to IFO1802 assembly (Scannell et al., 2011) which was Illumina sequenced. Except chromosomes 7,12 and 16 all chromosomes were assembled in single contigs with end-to-end coverage.

|  | *S. kudriavzevii* IFO1802 | *S. kudriavzevii* CR85 |
| --- | --- | --- |
| Technology | Illumina | Nanopore |
| #Scaffolds/contigs | 1455 | 20 |
| N50 (Mbp) | 0.151 | 0.860 |
| Total assembly size (Mbp) | 11.7 | 11.9 |

**Supporting figures**

# Figure S1

**
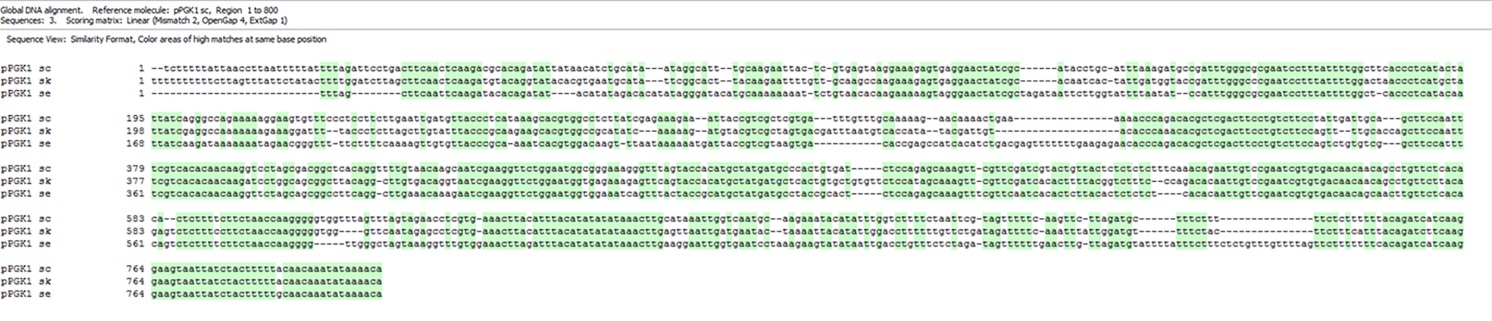
Alignment of the *S. cerevisiae, S. kudriavzevii* and *S. eubayanus PGK1* promoters.**

800 bp directly upstream of the ORF were used in the alignment. Green indicates nucleotides conserved in all three promoters

# Figure S2

**Impact of oxygen availability and respiratory growth on mRuby2 fluorescence signal.**

Optical density and fluorescence intensity of strain IMX1097 expressing *mRuby2* from the *SeFBA1p* during growth in chemically defined medium with glucose (SMG) (A,B) and ethanol (C,D) as carbon source in 96-well plates. Cultures with intact seal are indicated in grey, and cultures with punctured seals are indicated in black. Two independent culture replicates are shown for each condition. During growth on ethanol there is a strict requirement for oxygen, but no limitation was observed in growth and fluorescence of the strains (C,D)


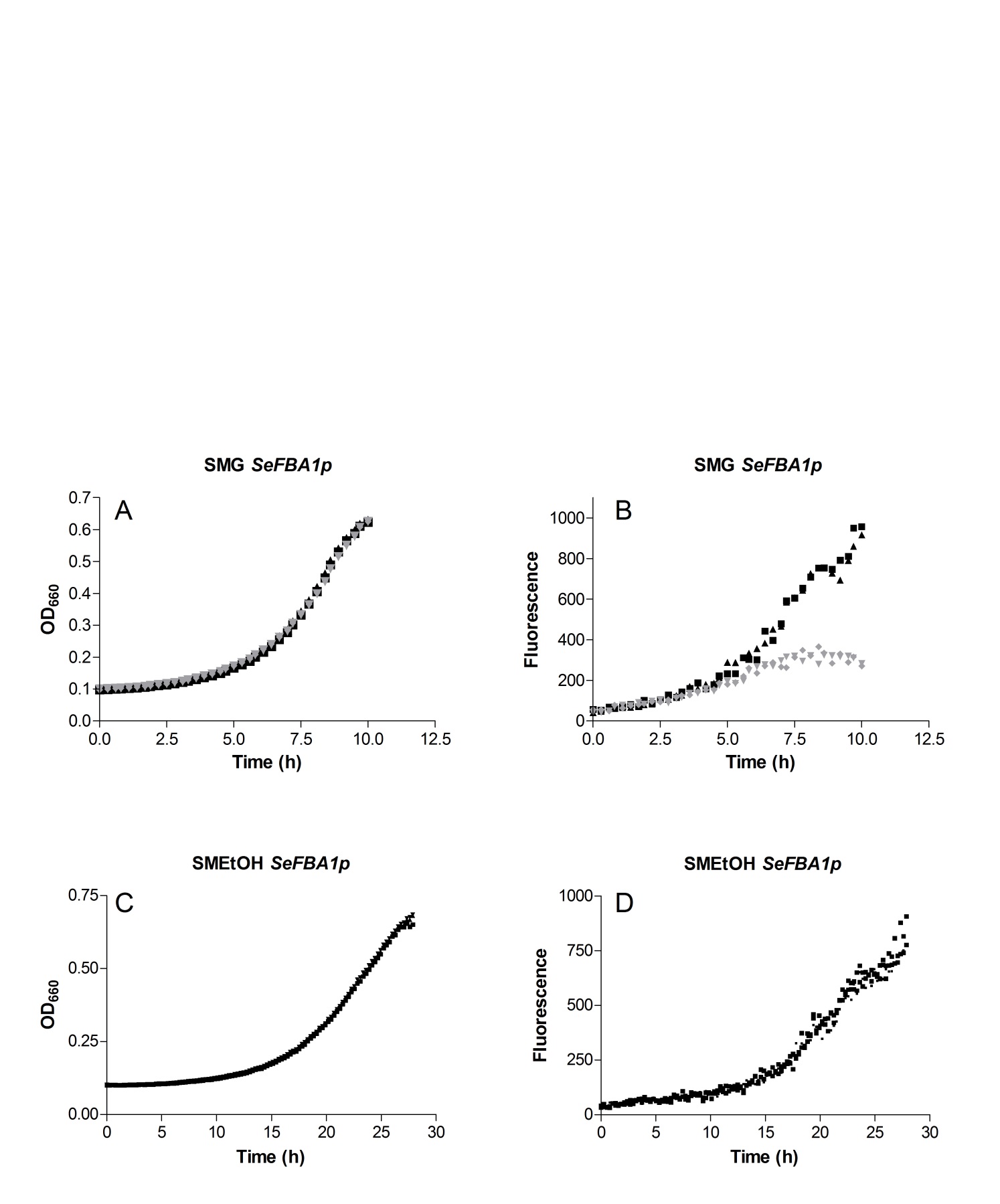


# Figure S3

**Comparison of fluorescence intensity measurements by plate reader and by flow cytometry.**

**
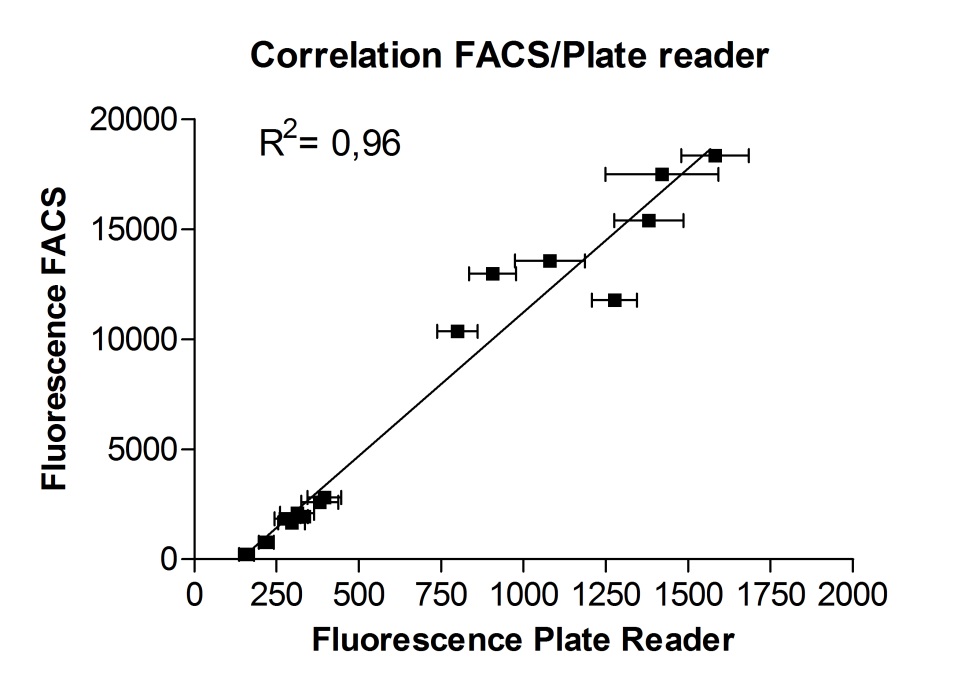
**Each data point represents the average fluorescence of five culture replicates from the same plate measured by the plate reader and the average fluorescence measurement of 10000 cells of the same cultures by flow cytometry, for a single strain. Results for 15 strains expressing mRuby2 with different promoter are shown. Error bars represent the SEM.

# Figure S4

**
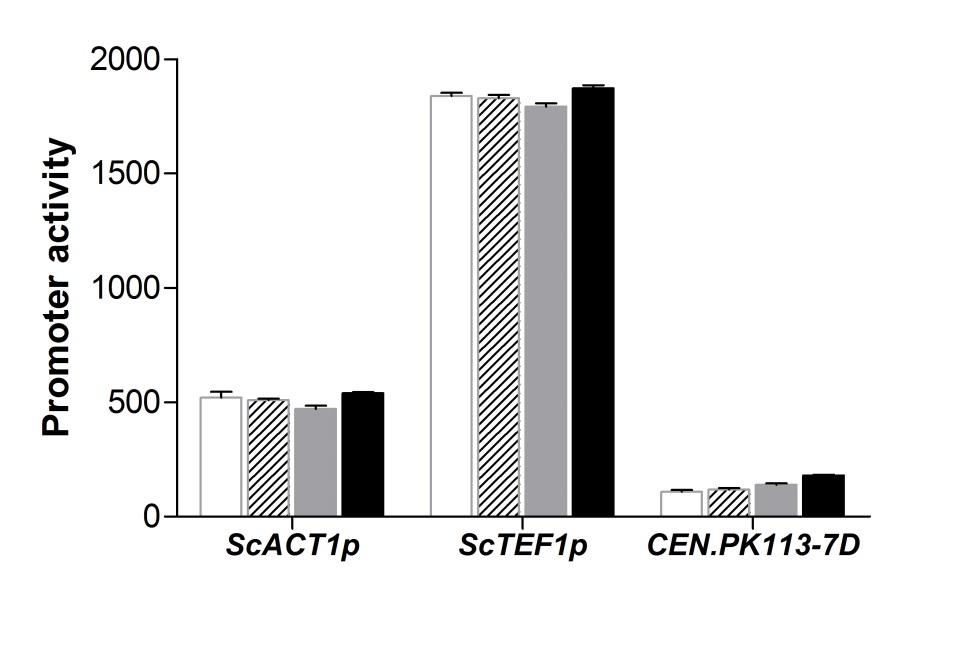
Reproducibility of fluorescence measurements between 96-well plates.**

Fluorescence of strains expressing *mRuby2* driven by *S. cerevisiae ACT1* and *TEF1* promoters, and background fluorescence of the prototrophic non-fluorescent control strain CEN.PK113-7D. For each strain, the four bars represent the fluorescence measurements of four independent plate cultures. Bars and error bars represent the average and standard deviation of the mean of 6 biological replicates from the same plate, respectively

# Figure S5

**Promoter strength characterization by flow cytometry.**

**
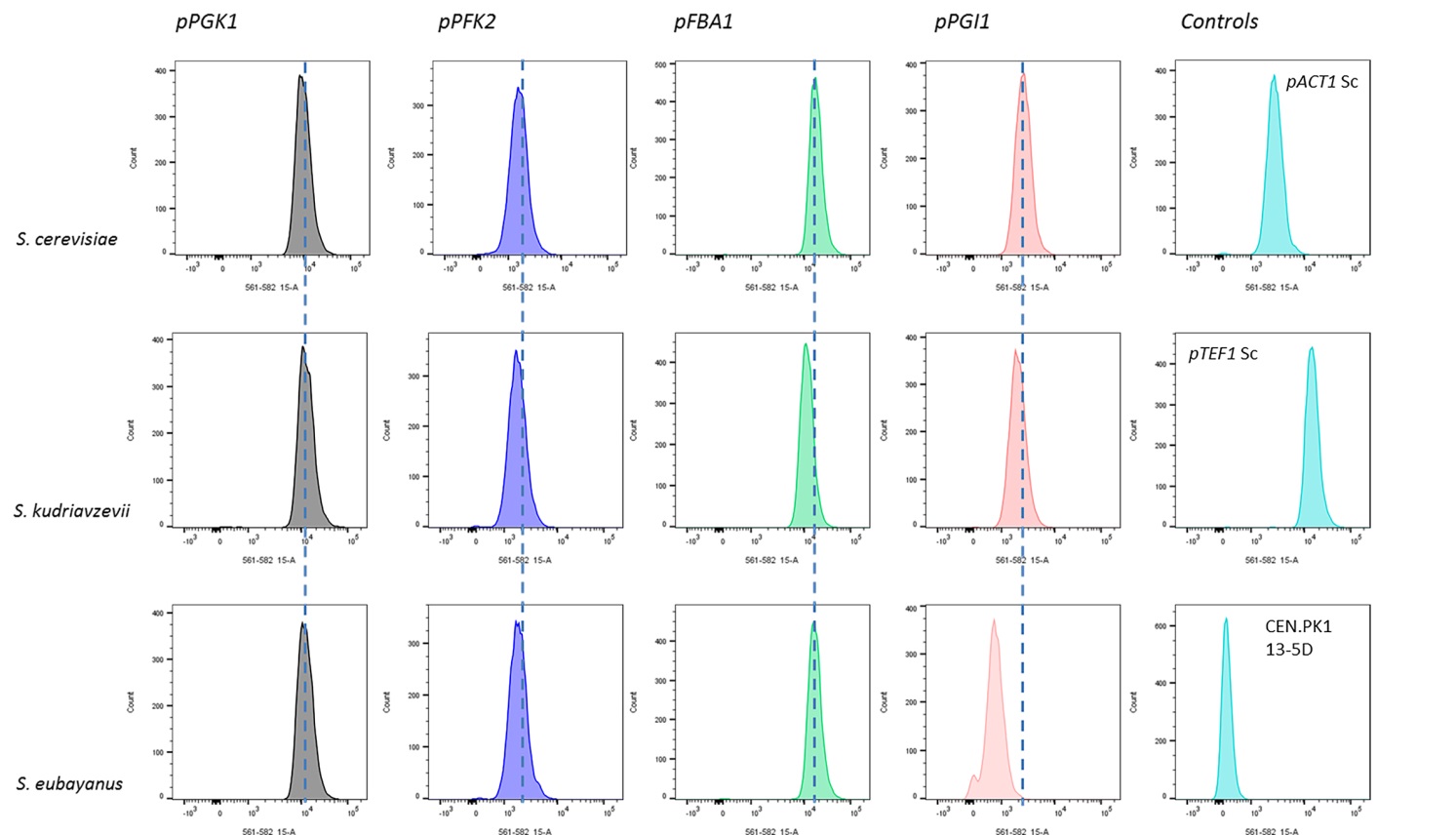
**The fluorescence of cells from exponentially growing cultures from a 96-well plate containing promoter-*mRuby2* reporter constructs was analysed by flow cytometry. For each strain the fluorescence profile for 10000 cells is shown. Strains expressing mRuby2 with the *ScACT1* and *ScTEF1*promoters and CEN.PK113-5D not expressing mRuby2 were taken as controls.

# Figure S6

**Influence of growth conditions on the promoter strength of strains from the *S. cerevisiae* library expressing mRuby2 under the control of various glycolytic promoters.**

*S. cerevisiae* strains with glycolytic promoters from *S. cerevisiae* (triangle), *S. kudriavzevii* (diamond) and *S. eubayanus* (circle)*.* Fluorescence was measured from whole cultures in plate reader.


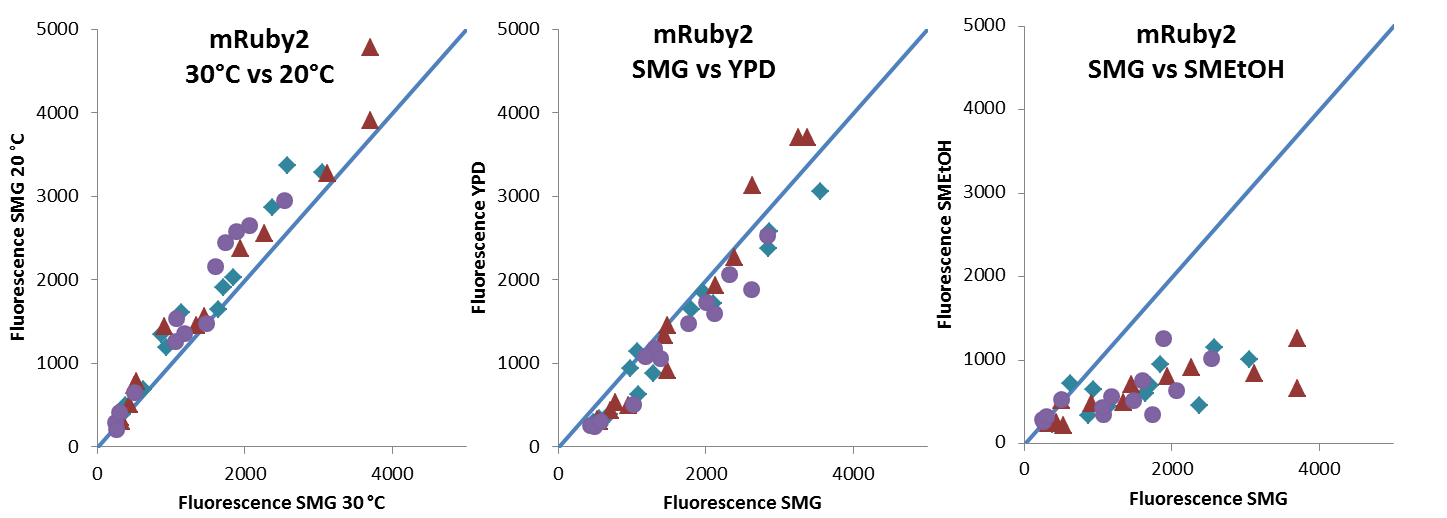


# Figure S7

**Comparison of transcription measured by RNAseq and fluorescent reporter.**

RNAseq data were normalized to *ACT1* transcript level and mRuby2 data to the fluorescence levels driven by the *ACT1* promoter.

**
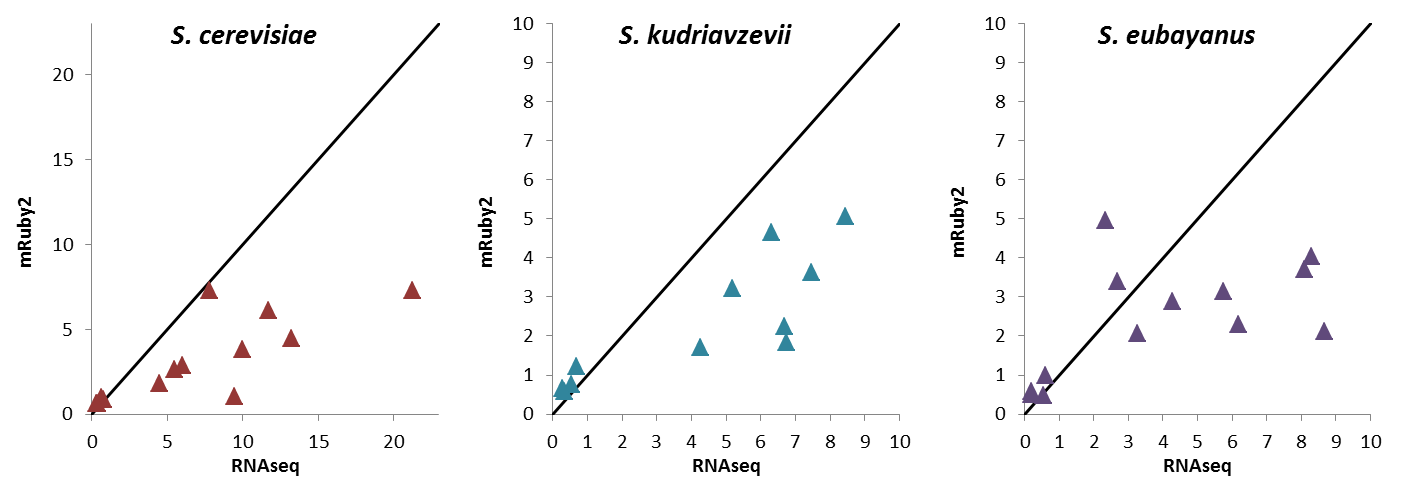
**

# References

Lee, M.E., DeLoache, W.C., Cervantes, B., and Dueber, J.E. (2015). A highly characterized yeast toolkit for modular, multipart assembly. *ACS Synth. Biol.* 4**,** 975-986. doi: 10.1021/sb500366v.

Scannell, D.R., Zill, O.A., Rokas, A., Payen, C., Dunham, M.J., Eisen, M.B., et al. (2011). The awesome power of yeast evolutionary genetics: new genome sequences and strain resources for the *Saccharomyces* sensu stricto genus. *G3: Genes, Genomes, Genetics* 1**,** 11-25. doi: 10.1534/g3.111.000273.
